# microRNAs Mediated Regulation of the Ribosomal Proteins and its Consequences on the Global Translation of Proteins

**DOI:** 10.3390/cells10010110

**Published:** 2021-01-08

**Authors:** Abu Musa Md Talimur Reza, Yu-Guo Yuan

**Affiliations:** 1 Jiangsu Co-Innovation Center of Prevention and Control of Important Animal Infectious Diseases and Zoonoses, College of Veterinary Medicine, Yangzhou University, Yangzhou 225009, China; talimurku@gmail.com; 2 Institute of Biochemistry and Biophysics, Polish Academy of Sciences, Pawińskiego 5a, 02-106 Warsaw, Poland; 3 Jiangsu Key Laboratory of Zoonosis/Joint International Research Laboratory of Agriculture and Agri-Product Safety, The Ministry of Education of China, Yangzhou University, Yangzhou 225009, China

**Keywords:** Ribosomal proteins (RPs), microRNA (miRNA), ribosomes, translation, protein synthesis

## Abstract

Ribosomal proteins (RPs) are mostly derived from the energy-consuming enzyme families such as ATP-dependent RNA helicases, AAA-ATPases, GTPases and kinases, and are important structural components of the ribosome, which is a supramolecular ribonucleoprotein complex, composed of Ribosomal RNA (rRNA) and RPs, coordinates the translation and synthesis of proteins with the help of transfer RNA (tRNA) and other factors. Not all RPs are indispensable; in other words, the ribosome could be functional and could continue the translation of proteins instead of lacking in some of the RPs. However, the lack of many RPs could result in severe defects in the biogenesis of ribosomes, which could directly influence the overall translation processes and global expression of the proteins leading to the emergence of different diseases including cancer. While microRNAs (miRNAs) are small non-coding RNAs and one of the potent regulators of the post-transcriptional gene expression, miRNAs regulate gene expression by targeting the 3′ untranslated region and/or coding region of the messenger RNAs (mRNAs), and by interacting with the 5′ untranslated region, and eventually finetune the expression of approximately one-third of all mammalian genes. Herein, we highlighted the significance of miRNAs mediated regulation of RPs coding mRNAs in the global protein translation.

## 1. Introduction

MicroRNAs (miRNAs) and Ribosomal proteins (RPs) are two important classes of regulatory molecules that control the translation of proteins from messenger RNA (mRNA). RPs are highly conserved proteins across all the forms of life [1], at least 53 RPs are detected in Escherichia coli and 80 RPs are detected in mammals [2], which are active structural components of ribosomes, the machinery and master regulators of the protein translation process. Deregulation of RPs could interfere with the overall translation process by generating ribosome heterogeneity (‘specialized ribosomes’), which may change the global protein synthesis and/or favor the translation of a subset of proteins [3,4,5]. While miRNAs is a class of non-coding RNAs that can interfere with the protein translation process by targeting the 3′-untranslated region and/or coding region of mRNAs [6] as well as interacting with the 5′ untranslated region [7] and eventually the expression of approximately one-third of all mammalian genes, which are finetuned by the miRNAs [8]. Similarly, the expressions of RPs are also finetuned by the miRNAs binding to their transcripts following the facilitation or restriction of the translation process [7,9,10,11]. The miRNAs involved in the regulation of RPs coding mRNAs are eventually regulating the global translation of proteins through its subsequent impact on the biogenesis of ribosomes as well as the assembly and formation of the translation machinery [7]. Therefore, understanding this group of miRNAs required extra attention and will improve our understanding of the multi-dimensional interactions among miRNAs, RPs, biogenesis of ribosomes and global gene expressions.

Several excellent reviews have focused on the biogenesis of miRNAs [12,13,14,15] and their regulatory roles on the overall process of translation [10,11,16,17,18]. Those reviews are excellent resources in order to develop an understanding of the regulatory roles of miRNAs in gene expression. However, the understanding of miRNAs mediated regulation of RPs coding mRNAs is still in the primary stage, and literally very few original researches have been published in this context. Therefore, an overview outlining the potential and importance of investigating the regulatory role of miRNAs on the Ribosomal protein coding mRNAs is required. Herein, we summarize the interaction and networking among the miRNAs, RPs, ribosome biogenesis and global gene expression, and their direct and indirect influence on the disease progression. Firstly, we discuss the regulatory roles of RPs on the biogenesis of ribosomes, assembly and formation of translation machinery, translation of the proteins and the progression of diseases. Secondly, we discuss the regulatory roles of miRNAs on the translation and expression of the RPs, and the interaction and networking between miRNAs and RPs. Finally, we discuss the cumulative and interactive influence of the miRNAs and RPs on disease progression and provide conclusive remarks to conduct future research on this topic.

## 2. Materials and Methods

Published related articles were searched for using keywords: Ribosomal proteins (RPs), miRNAs, gene expression, translation, post-transcriptional regulation of mRNA and ribosomopathy in the National Center for Biotechnology Information PubMed database up until November 2020. Relevant articles published in English were included in this review. We only focused on the RPs and the diseases related to the deregulation of RPs, and the miRNAs targeting RPs. Human RPs were categorized according to their involvement in different biological processes using the DAVID bioinformatics resources (https://david.ncifcrf.gov/). The genetic alteration status of the 73 RPs coding genes, and its consequences in different types of cancer patients were analyzed using the cBioPortal for Cancer Genomics (http://www.cbioportal.org/index.do) database from the cancer genome atlas (TCGA) Research Network (https://www.cancer.gov/about-nci/organization/ccg/research/structural-genomics/tcga). For analysis, all samples (10,967 samples) in “TCGA PanCancer Atlas Studies (32 categories)” were included and analyzed as a group for checking the genetic alteration (both mutation and copy number alteration) and overall survival status of the patients, the bookmark link of the analysis is collected for future reference or revisit (http://bit.ly/2YsULwu). The expression pattern of 73 RPs coding mRNA (heatmap) in “serous ovarian cancer (TCGA PanCancer Atlas)” samples was also generated from the cBioPortal database, and the bookmark link was collected for future reference or revisit (http://bit.ly/2Lxitmg), in which 300 clinical ovarian cancer patients samples having mRNA expression data (RNA Seq V2) were taken into consideration. The interaction between RPs and miRNAs was predicted using miRNet database (https://www.mirnet.ca/), which is an integrated platform to link the miRNAs to their target mRNAs and their functions.

## 3. RPs on the Biogenesis and Assembly of Ribosomes, and Translation of Proteins

A ribosome is a supramolecular ribonucleoprotein complex [19], composed of Ribosomal RNA (rRNA) and RPs, and coordinates the translation and synthesis of proteins with the help of transfer RNA (tRNA) and other factors [19]. Increase or decrease in the biogenesis of ribosomes directly influence the translation process and global gene expression following the growth, proliferation and differentiation of the cells [19,20,21,22], as well as physiological processes and progression of diseases [20,23,24,25]. RPs are mostly derived from the energy-consuming enzyme families such as ATP-dependent RNA helicases, AAA-ATPases, GTPases and kinases [26] and are involved in a vast array of biological processes (Table 1). Although RPs are known for their inevitable role in the biogenesis and assembly of translation machinery (Table 2), not all RPs are indispensable for functional ribosomes. The ribosome complex could be functional and continue the translation of proteins instead of lacking in some RPs [27], but the efficacy and accuracy of protein synthesis might be compromised. For example, Ribosomal protein L33 (RPL33) is required for ribosome biogenesis, subunit joining, and a mutation in RPL33 causes the repression of GCN4 translation in yeast [28], which is a transcription factor and master regulator of the genes, and is highly conserved in mammalian species named as activating transcription factor-4 (ATF4) [29]. The mutation in RPL33 also reduces the processing efficiency of the 35S and 27S pre-rRNAs following the reduction of the accumulation of all four mature rRNAs [28]; thus, RPL33 could have a mass influence on the global gene expression through the deregulation of master transcription factor GCN4 or ATF4. Similarly, Ribosomal protein S20 (RPS20) is responsible for the mRNA binding and subunits association, and lacking RPS20 causes drastic reduction in the formation of the 70S complex as well as mRNA binding through an initiation that defects to the 30S subunit [30]. Ribosomal protein L16 (RPL16) is required for the assembly of 60S subunits, and Ribosomal protein 59 (RP59) is required for the assembly of the 40S subunit [31], while Ribosomal protein L1 (RPL1) plays essential roles in maintaining the stability of 5S rRNA as well as the assembly of 60S subunits in yeast [32,33] and Ribosomal protein L9 (RPL9) is essential for the small subunit maturation in E. coli bacteria [34]. Therefore, many of the RPs are eventually essential for the proper biogenesis, assembly and functioning of the translation machinery.

In addition to the assembly of ribosomes, RPs play important accessory roles to facilitate the biosynthesis and post-translational modification of proteins such as processing and folding of rRNA, assembly and transportation of the precursors of ribosomes, stabilization of the Ribosomal subunits [35], enzymatic activities [36], as well as folding [37,38] and co-translational translocation [39,40] of the proteins. Take, for example, the Ribosomal protein L23 (RPL23), which is a docking site for a chaperone on the ribosome, and has a regulatory role on the chaperone-assisted folding of proteins [37], RPL23a along with Ribosomal protein L35 (RPL35) also showed an important role during peptide recognition and insertion to the translocation channel by repositioning SRP54 [38]. Similarly, Ribosomal protein S12 (RPS12), Ribosomal protein S4 (RPS4), Ribosomal protein S9 (RPS9) and Ribosomal protein S28 (RPS28) are important for translational accuracy [41,42,43,44], while Ribosomal protein L3 (RPL3), Ribosomal protein L5 (RPL5), Ribosomal protein L24 (RPL24), Ribosomal protein L39 (RPL39) and Ribosomal protein L41 (RPL41) potentially influence the peptidyltransferase activity and subunit association of the ribosomes [45,46,47]. Furthermore, RPL5 regulates the anchoring of the peptidyl-tRNA to the P-site in Yeast [47], and Ribosomal protein L10 (RPL10) plays an important role in the nuclear export by interacting and releasing cytoplasmic Nmd3p from the 60S subunit [48,49,50]. The interaction between Ribosomal protein L1 (RPL1) and Ribosomal protein L16 (RPL16) is required for the stabilization of 5S rRNA, while Ribosomal protein L12 (RPL12) mediates the correct assembly of ribosomal stalk [32,33,51,52]. Ribosomal protein S14 (RPS14), Ribosomal protein S0 (RPS0) and Ribosomal protein S21 (RPS21) are involved in the cytoplasmic rRNA processing steps leading to the maturation of 18S rRNA [53,54,55]. Ribosomal protein L25 (RPL25) is required for pre-rRNA processing [56]. The Ribosomal protein S15 (RPS15) is required for a nuclear exit of the 40S subunit precursors in yeast [57] and Ribosomal protein S14 (RPS14) is required for the maturation of 43S pre-ribosomes [53]. Ribosomal protein S12 (RPS12) increases the rate of translation with the cost of high rate of error in the protein synthesis, while Ribosomal protein S4 (RPS4) and Ribosomal protein S5 (RPS5) are required to maintain the accuracy of protein translation [58,59,60]. Therefore, RPs are an integral part of the translation machinery, and many of them play an inevitable role during the biogenesis of ribosomes and the translation process, while few of them might be dispensable for a functioning translation machinery but must have its consequences.

## 4. RPs Mediated Regulation of Biological Processes and Progression of Diseases

RPs are not simply static building blocks of the ribosome; they are critical regulators of different biological processes (Table 1), important components of cellular organelles (Supplementary Table S1) and are involved in various molecular functions (Supplementary Table S2) of the cells. RPs are directly and indirectly involved in the various important molecular signaling pathways such as RP-MDM2-p53 signaling [62,63], which are involved in the regulation of diverse physiological processes including energy metabolism to the growth and proliferation of the cells. Hence, deregulation of RPs impairs the synthesis, processing and assembly of rRNA, translation and modification of proteins, and eventually lead to the progression of diseases (Table 3) including developmental, systemic and metabolic complications, and cancers [24,62,63,64,65,66]. The diseases that are derived from the structural and functional defects of the RPs or rRNA genes or the genes in which products are involved in the assembly and biogenesis of ribosomes are defined by the term ribosomopathy [67,68,69]. The diseases that come under the term ribosomopathy include Diamond-Blackfan anemia (DBA), 5q-syndrome, Schwachman-Diamond syndrome (SDS), X-linked dyskeratosis congenita (DC), cartilage hair hypoplasia (CHH), Treacher Collins syndrome (TCS), Bowen-Conradi syndrome (BCS), North American Indian childhood cirrhosis (NAIC) [67].

DBA is characterized by anemia, retardation of growth and congenital deformities, and could be a result of the structural and functional defects of 10–15 ribosomal proteins including RPS19, RPS26, RPL5 and RPL11 [70,71,72]. 5q-syndrome is a type of anemia that is caused by the haplo-insufficiency of RPS14 [73], a critical component for 40S assembly, and depletion of RPS14 in human CD34+ cells is sufficient to recapitulate the 5q-defect of erythropoiesis with sparing of megakaryocytes [91]. The clinical sign of SDS disease includes exocrine pancreatic insufficiency, hematologic abnormalities such as neutropenia, neurocognitive dysfunction [92,93,94], and results from the bi-allelic mutations in the ribosome maturation protein SBDS, which compromises its ability to couple GTP hydrolysis by the GTPase EFL1 to the release of eIF6 from the 60S subunit [74]. DC is the X-linked subtype of dyskeratosis congenita, and the symptoms include mucocutaneous abnormalities such as pigmented skin, changes of nail, failure of bone marrow and pulmonary fibrosis [75]. CHH is characterized by the short stature deformities of bone and abnormalities in the growth of hair and potentially results from mutation of the RMRP gene [76]. TCS is identified by the craniofacial abnormalities and caused by the mutation in the TCOF1 gene [77], which is involved in rRNA transcription. BCS is the result of an autosomal recessive abnormality of the EMG1 gene, which plays a role in small ribosomal subunit assembly [78,79,80]. NAIC is an autosomal recessive abnormality of the CIRH1A gene, which codes for cirhin, and clinical symptoms include biliary, cirrhosis, portal and hypertension [81,82].

**Figure 1:**
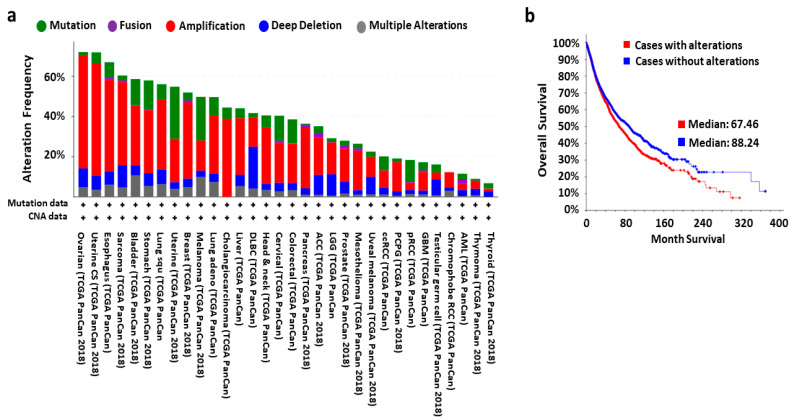
Alteration status of ribosomal proteins (RPs) in cancers and its correlation with patient’s survival status. (**a**) Bar diagram showing the copy number alteration status of RPs in different types of cancers, the data obtained by analyzing 10,967 samples from “TCGA PanCancer Atlas Studies (32 categories)”, it showed that all of the 32 cancer types have altered the expression of RPs, while ovarian cancer, uterine cancer and esophagus are the top three types of cancer, of which more than 60% samples have alteration of RPs. (**b**) Survival graph showing that altered expression of RPs are negatively correlated with the survival status of cancer patients. All data were generated using the cBioPortal for Cancer Genomics (http://www.cbioportal.org/index.do) database from the cancer genome atlas (TCGA) Research Network (https://www.cancer.gov/about-nci/organization/ccg/research/structural-genomics/tcga).

The clinical patient data obtained from the cBioportal database (https://www.cbioportal.org/) of the TCGA research network showed the frequent copy number alteration of the RPs coding genes (Figure 1a). It also showed that the patients with an alteration status of RPs had lower median month survival compared to the patients without alterations in the RPs (Figure 1b). Moreover, deregulated expression of the RPs is also recorded at the mRNA level, for example, the RNA-seq data of 300 ovarian cancer patients obtained from the same database (Supplementary Figure S1). The deregulated expression of the RPs in different types of cancers is also supported by the published research findings [95,96,97,98], such as breast cancer [99,100], gastric cancer [101], hepatocellular cancer [102], colorectal cancer [103,104,105], prostate cancer [66,106], and the expression of RPs varies between normal and malignant cells as well as across the types of cancers [107]. For example, downregulation of Ribosomal protein S6 (RPS6) inhibits the growth of non-small cell lung cancer by inducing cell cycle arrest, rather than apoptosis [83], X-linked ribosomal protein S4 (RPS4X) is an independent prognostic factor in patients with serous epithelial ovarian cancer, and the low expression of RPS4X is associated with a poor prognosis in human serous epithelial ovarian cancer [85] and bladder cancer [86], RPL31 is overexpressed in prostate carcinomas compared with benign prostate tissues, and Ribosomal protein L31 (RPL31) might promote the growth of prostate cancer cell by increasing the degradation of tumor suppressor p53 [87]. Ribosomal Protein L34 (RPL34) functions as an oncogene and modulates esophageal cancer cells by the inactivation of the PI3K/Akt signaling pathway, and silencing of RPL34 inhibits the proliferation and metastasis of esophageal cancer cells [88]. Ribosomal Protein L22 (RPL22) controls the dissemination of T-cell lymphoma: single copy loss of RPL22 promoted lymphomagenesis and dissemination, while loss of both copies results in mediastinal retention [89]. Mutation of Ribosomal Protein S20 (RPS20) in the germline cells might cause hereditary nonpolyposis colorectal carcinoma [90]. Based on the above discussion, the RPs are not only the building block of the translation machinery, but also have important roles in other physiological processes, and deregulation of the RPs might cause severe diseases including different types of ribosomopathy and cancers.

## 5. MicroRNAs Mediated Regulation of Gene Expression and Progression of Diseases

miRNAs are small non-coding RNAs (~22 nucleotides in length) and are involved in RNA silencing [14]. miRNAs are one of the potent regulators of the post-transcriptional gene expression, which regulate gene expression by targeting the 3′ untranslated region and/or coding region of the mRNAs [6], and also by interacting with the 5′ untranslated region [7], and eventually finetune the expression of approximately one-third of all mammalian genes [8]. These findings have established the fact that miRNAs are an inevitable mediator of health and diseases in both humans and animals [108,109]. Many publications reported the involvement of miRNAs in almost all bio-physiological processes starting from the germ cells [109] to the development of the nervous system [110], immune regulation [111,112,113], and proliferation and differentiation of cells [114,115,116,117,118]. As well, miRNAs are reported to be involved in numerous human and animal diseases: for example, miRNAs are detected as both oncomir and tumor suppressors, and their roles varied depending on the miRNA-family as well as the type of cancer [119,120,121,122,123,124,125,126]. However, there is a prominent trend of global suppression of miRNAs expression in different types of cancers [127], and this process of global suppression of miRNAs expression could be a result of multiple conditions such as genomic defects in the miRNA coding region (mutations, amplifications or deletions), transcription factor mediated repression (such as Myc), epigenetic alterations in the promoter region (CpG islands hypermethylation), and the deregulation of Dicer and Drosha, the machineries for the processing of miRNAs [127].

Deregulated expression of miRNAs is detected with many other types of diseases such as neurodegenerative diseases including Alzheimer’s, Parkinson’s, Huntington’s disease [128,129] and amyotrophic lateral sclerosis [130], eye diseases including glaucoma, and myopia [129,131], traumatic brain injury [132], diabetes [133], rheumatoid arthritis [134,135], autoimmune and chronic inflammatory diseases [136], lung diseases [137], skeletal diseases [138], age-related diseases [139,140], myocardial infarction and cardiovascular diseases [141,142]. In addition, certain physiological abnormalities, such as hypoxia, which is a reduction in the normal tension of tissues oxygen (O_2_) level, and a characteristics feature of chronic vascular and pulmonary disease and many cancers, resulted in a deregulated expression of miRNAs [143], and several hypoxia induced miRNAs play important roles in the adaptation of cancer cells to the hypoxia [143]. miRNAs are also involved in the immune suppression process and the potential use of miRNAs manipulation strategy for prolonging the immune tolerance following the survival of allograft is also evident through preclinical studies [144]. Therefore, proper understanding of miRNAs related regulation of health and diseases will clarify the ways to develop treatment strategies and preventive measures against many fatal diseases.

## 6. MicroRNAs Biogenesis and Dissemination to the Circulatory System

**Figure 2:**
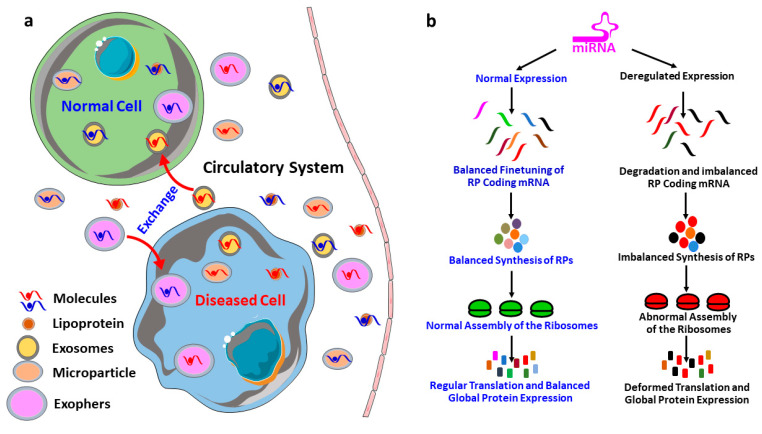
Potential mechanism of microRNA (miRNA) mediated regulation of global protein synthesis. (**a**) Cell-cell and cell-stromal crosstalk: the transportation of the cell secreted miRNAs and proteins by the extracellular vesicles (exosomes, microparticle and exophers) and lipoproteins to the circulatory system, which carries them further to the neighboring cells and the distant organs. (**b**) Deregulated expression of miRNAs might cause an imbalance in the expression of ribosomal proteins (RPs) followed by the abnormal assembly of the protein translation machinery (ribosomes); therefore, the ultimate outcome would be the deregulation of the global protein synthesis.

By regulating, finetuning and silencing of the protein-coding transcripts, miRNAs play an inevitable role in gene expression, molecular signaling and pathological conditions of different diseases. There are excellent reviews that explain every aspect of miRNAs biogenesis [12,13,14,15]. However, the dissemination of miRNAs to the circulatory system and its role in the cell–cell and cell–stromal crosstalk need to be explained here for a proper understanding of the discussed topic in this review. As shown in Figure 2a, cells (under both normal and diseased conditions) produce different kinds of extracellular vesicles including the exosomes [120], microvesicles [145] and exophers [146], which are used to expel the waste material outside of the cells. These extracellular vesicles contain protein, miRNAs, lipids and other waste material derived from the originating cells. Therefore, these vesicles are excellent biomarkers to study the pathological conditions of the originating cells. In addition to dumping the waste materials of the cells, these vesicles containing the proteins and miRNAs from the originating cells could be carried by the circulatory system to the neighboring cells as well as to the distant tissues, and thereby participate in the cell–stromal and cell–cell communication. Therefore, the miRNAs do not only influence the gene expression of its mother cells, but it can also influence the gene expression and transcript silencing of the neighboring cells and distant tissues. This signifies the role of miRNAs in the progression and dissemination of diseases to the secondary organs, which means organs other than the organ of the disease outbreak.

## 7. MicroRNAs in the Regulation of Ribosomal Protein Coding mRNAs

miRNAs are involved in the regulation and finetuning of at least one third of all mammalian genes [8], and thus, it is not unusual to anticipate that miRNAs are also interfering with the post-transcriptional expressions of most of the ribosomal proteins coding mRNAs and eventually finetuning the overall protein synthesis. To illustrate, firstly, the deregulation in the expression of miRNAs, which are involved in the regulation of RPs coding mRNAs, could result in an imbalanced synthesis of RPs. Secondly, an imbalance in the expression of RPs could lead to the defective assembly and biogenesis of the ribosomes, and/or functional abnormality to the translation machinery [19,20,21,22]. Finally, the structural and functional abnormality of the ribosomes results in an inefficient and atypical translation, and eventually influences the global translation of proteins (Figure 2b) followed by the physiological abnormalities and progression of diseases [20,23,24,25]. Take, for example, miR-10a, which is reported to positively influence the global translation of proteins by interacting with the 5′ untranslated region of the ribosomal protein coding mRNAs [7]. This signifies that the RPs regulatory miRNAs could be considered as master miRNAs, which might be a small group but potentially interferes with the global expression of the genes. Therefore, understanding this group of miRNAs required extra attention, and will improve our understanding of the multi-dimensional interactions among miRNAs, RPs, biogenesis of ribosomes and global gene expressions. However, the investigation about the role of miRNAs on the RPs are very limited, the very few findings in this field includes: miR-7641 potentially play a role in cancers through the regulation of ribosomal protein S16 (RPS16) [9], miR-10a positively influences the global translation of proteins by interacting with the 5′ untranslated region of the ribosomal protein coding mRNAs [7], and miR-147b inhibits the proliferation and invasiveness of the non-small cell lung cancer (NSCLC) by downregulating the RPS15A mediated signaling of the Wnt/β-catenin [84].

**Figure 3:**
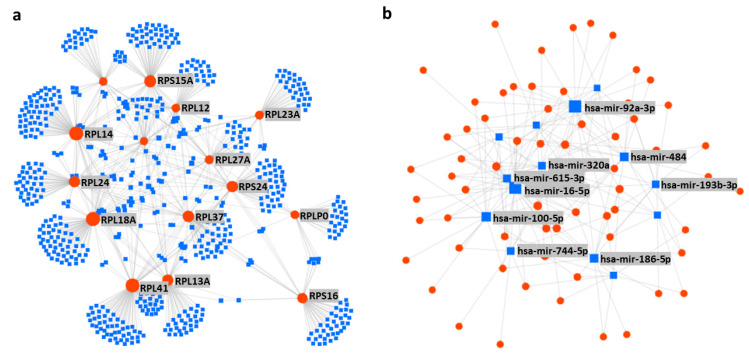
Interaction network between ribosomal proteins (RPs) and microRNAs (miRNAs). (**a**) Top 15 RPs are grouped based on their number of connections to the miRNAs (RPL41, RPL14, RPL18A, RPS15A, RPL13A, RPL24, RPS24, RPL37, RPS16, RPL12, RPS27A, RPS19, RPL27A, RPL23A, and RPLP0, respectively), and these 15 RPs are predicted to be connected by 670 miRNAs. (**b**) Top 14 miRNAs are grouped based on their number of connections with the RPs (hsa-mir-16-5p, hsa-mir-92a-3p, hsa-mir-100-5p, hsa-mir-615-3p, hsa-mir-484, hsa-mir-186-5p, hsa-mir-320a, hsa-mir-193b-3p, hsa-let-7a-5p, hsa-mir-331-3p, hsa-mir-92b-3p, hsa-mir-652-3p, hsa-mir-766-3p, and hsa-mir-744-5p, respectively), and these 14 miRNAs are potentially connected with 66 RPs. The size of the nodes indicates the degree of connectivity; the bigger the node size, the higher the connection. The interaction network was generated using the miRNet database (https://www.mirnet.ca/).

Now, two things are very clear: (1) there are master miRNAs that can influence the global translation of proteins [7], and (2) the number of investigations is very small to understand miRNAs mediated regulation of RPs. However, a vast network between the RPs and miRNAs is predicted by the in-silico analysis (Supplementary Figure S2), which shows more than a thousand miRNAs are potentially involved in the regulation of RPs. To find out the most vulnerable RPs to the miRNAs attack, the top 15 RPs were sorted based on their number of connections to the miRNAs, which include RPL41, RPL14, RPL18A, RPS15A, RPL13A, RPL24, RPS24, RPL37, RPS16, RPL12, RPS27A, RPS19, RPL27A, RPL23A, and RPLP0, respectively (Figure 3a); these 15 RPs are predicted to be connected with around 800 miRNAs (Supplementary Table S3). On the other hand, to distinguish the most important miRNAs, the top 14 miRNAs were grouped based on their number of connections with the RPs, these include hsa-mir-16-5p, hsa-mir-92a-3p, hsa-mir-100-5p, hsa-mir-615-3p, hsa-mir-484, hsa-mir-186-5p, hsa-mir-320a, hsa-mir-193b-3p, hsa-let-7a-5p, hsa-mir-331-3p, hsa-mir-92b-3p, hsa-mir-652-3p, hsa-mir-766-3p, and hsa-mir-744-5p, respectively (Figure 3b). These 14 miRNAs are potentially connected with most of the RPs and are reported to be involved in many diseases including different types of cancers (Table 4). A brief review about the above-mentioned 14 miRNAs is performed to understand the already known roles of those miRNAs, in terms of their involvement in the biophysiological processes and progression of diseases.

The top connected miRNA to the RPs is miR-16-5p, which is an important miRNA, plays a role in the proliferation and differentiation of cells [266], and regulates different types of cancers, such as breast cancer cells by targeting VEGFA [147] and restraining the AKT3 mediated NF-κB pathway [148], hepatocellular carcinoma (HCC) by targeting IGF1R [149], mesothelioma by targeting CCND1 and BCL2 [150], glioma by targeting the cell cycle and apoptotic mediators [151], neuroblastoma by targeting MYCN [152], as well as regulates chordoma, gastric cancer and osteoarthritis by targeting SMAD3 [153,154,155] and provides protection against LPS-induced cell injury by targeting CXCR3 [267]. Furthermore, miR-16-5p might have a role in osteoclastogenesis [156] and could be an important biomarker of rheumatoid arthritis [157]. This signifies that miR-16-5p is an active regulator of different biological processes, and further investigation is required, particularly to understand how it influences the expression of RPs, which has not been investigated.

The second top miRNA is mir-92a-3p, which has involvement in several pathological conditions: it is a potential oncomir [158,268], overexpression creates resistance to the TRAIL-dependent apoptosis by suppressing MYCBP2 in melanoma [158], promotes the progression of liposarcoma by stimulating the tumor-associated macrophages to secret IL6, a proinflammatory cytokine [159], promotes tumorigenesis in glioma cells by regulating cadherin 1 (CDH1)/β-catenin signaling but at the same time reduces the stemness of glioma stem cells (GSCs) by modulating Notch-1/Akt signaling [160]. Similarly, blocking of miR-92a-3p induces apoptosis in leukemia [161] and colorectal cancer cells [162,163]. Instead of its pro-cancer role, it prevents the degradation of cartilage by targeting WNT5A [164] and might be therapeutically significant for the treatment of osteoarthritis. It also has diagnostic value, miR-92a-3p is a biomarker for several diseases such as Kawasaki disease [165], schizophrenia [166], systemic lupus erythematosus [167], and white matter impairment and post-stroke depression [168]. However, the involvement of miR-92a-3p in the regulation of RPs is not investigated, which is important to understand its role in the regulation of translation machinery.

The third, fourth and fifth miRNAs in the list are miR-100-5p, miR-615-3p and miR-484, respectively: oncogenic miR-100-5p is a potent regulator of viability, metastasis and apoptosis of different cancer types, blocking of miR-100-5p induces apoptosis and prevents the re-emergence of prostate cancer [169], renal cell carcinoma (RCC) [170], oral cancer [171] and NSCLC [172]. It could also be used as a prognostic marker for HCC [173], abeta-induced pathologies [174] and hidradenitis suppurativa [175]. While, miR-615-3p plays important role in the differentiation of cells, it suppresses GDF5 and FOXO1 and inhibits osteogenesis in the lumbar ligamentum flavum cells [269], it is also known to have a feed-forward loop with HOXC5, and repress hTERT during the differentiation of cells [270]. In cancer, miR-615-3p plays a dual role depending on the type, for example, it promotes gastric cancer potentially by targeting CELF2 [176] and prostate cancer [177], but reported to be a suppressor of NSCLC potentially targeting IGF2 [178,179] and esophageal cancer [180]. In addition, it could be used as a biomarker for the recurrent HCC [181] and also regulates lipoapoptosis by targeting the C/EBP homolog in mice [271]. Similarly, miR-484 plays both ani- and pro-cancer role depending on the types, and it attenuates the epithelial to mesenchymal transition of cervical cancer by targeting ZEB1 and SMAD2 [182], metastasis by targeting MMP14 and HNF1A [183], and is usually downregulated in gastric cancer [184]. However, overexpression of miR-484 is considered as a poor prognosis factor for glioma patients, which targets MAP2 and activates ERK1/2 signaling resulting in the stemness characteristics of glioma cells [185]. miR-484 also promotes NSCLC by targeting APAF-1 [186] and adrenocortical cancer by targeting Fis1 [187], which are regulators of apoptosis. In addition, the presence of miR-484 in the blood serum could be considered as a biomarker for both NSCLC [272] and colorectal cancer [188]. As well, miR-484 promotes neurogenesis by targeting PCDH19 [273], prevents ischemia-reperfusion injury by inhibiting CAS3 and CAS9 mediated apoptosis of myocardial cells in rats [274], creates resistance to sunitinib mediated therapy in metastatic renal carcinoma [189] and reverses cytidine deaminase axis (CDA)-mediated chemoresistance in breast cancer [190].

The sixth and seventh most connected miRNAs to RPs are miR-186-5p and miR-320a, respectively: miR-186-5p is involved in several neurological and cardiac diseases such as the ischemia stroke, hippocampal neurons and coronary syndrome. In ischemia stroke, it targets IGF-1 that causes the apoptosis of neurons [191], while it regulates hippocampal neurons by controlling GLUA2 expression [192]. It prevents glucose-mediated injury of cardiomyocytes [275], potentially by regulating TLR3 [276] and could be a prognosis factor for acute coronary syndrome [193]. Furthermore, miR-186-5p regulates the secretion of FSH indicating its potential role in reproductive health [194]. In cancer, miR-186-5p showed both anti- and pro-cancer roles; for example, it shows anti-tumor properties in osteosarcoma by targeting FOXK1 [195] and TBL1XR1 [196], in colorectal cancer by targeting ZEB1 [197], in NSCLC by targeting SIX1 and in neuroblastoma by downregulating Eg5 [198], while it promotes lung adenocarcinoma by targeting PTEN [199] and metastatic prostate cancer [200]. On the other hand, miR-320a appears to be a global anti-cancer miRNA and reported to inhibit numerous types of cancers such as HCC by regulating HMGB1 expression [201,202], NSCLC by inhibiting the expression of ELF3 and inactivating PI3K/Akt signaling [203], gliomas by targeting SND1 and β-catenin [204], gastric cancer by targeting FOXM1-P27KIP1 [205] and RAB14 [206], lung adenocarcinoma by regulating STAT3 [207], tongue squamous cell carcinoma [208], multiple myeloma by inhibiting PBX3 [209], breast cancer by suppressing Rab14 [210], colorectal cancer by inhibiting RAC1 [211] and bladder carcinoma by directly inhibiting ITGB3 [212]. However, overexpression of miR-320a causes several non-cancer diseases including diabetic nephropathy by downregulating MafB [213], IL-1β-induced cartilage degradation by regulating PBX3 and NF-κB [214], osteoporosis [215] potentially by inhibiting MAP9 and PI3K/AKT signaling [216], doxorubicin-induced cardiotoxicity by targeting VEGF [217], anomalous placentation by targeting ERRγ [218], and atherogenesis by inhibiting SRF [219]. In addition, miR-320a could be a diagnostic tool for arrhythmogenic cardiomyopathy [220] and polycystic ovary syndrome [221].

The eighth, ninth and tenth of the candidate miRNAs are miR-193b-3p, let-7a-5p and miR-331-3p, respectively. Regulation of chondrogenesis by miR-193b-3p, potentially by the regulation of HDAC3, MMP19 and MMP16 is well documented [277,278,279]. It also contributes to preeclampsia by binding to the 3′UTR of TGFβ [222] and plays an anti-cancer role in several cancers, namely ovarian cancer by targeting PAK3 [223], breast cancer by regulating MORC4 [224], and urothelial cancer by targeting ETS1 and Cyclin D1 [225]. While, let-7a-5p inhibits osteogenesis and several cancers, the osteogenesis is inhibited by targeting TGFBR1 [226], and the lung cancer is inhibited by mediating G1/S phase arrest [227] most probably by regulating BCL2L1-mediated PI3Kγ signaling [228], it also inhibits HCC [229]. In addition, it could be a diagnostic marker for metastatic colorectal cancer [230] and might play an anti-apoptotic role in leukemia cells [231]. Among non-cancer diseases, let-7a-5p is involved in the pathogenesis of diabetic nephropathy by targeting HMGA2 [232], and could be a marker for hepatic fibrosis [233]. Similarly, miR-331-3p is also involved in different disease conditions, and known for both anti- and pro-oncogenic characteristics in different types of cancers: it promotes pancreatic cancer by targeting ST7L [234], HCC by downregulating E2F1 [235] and ING5 [236], while its’ presence in the serum indicates the invasive status of the HCC [237] as well as recurrence in the case of esophageal adenocarcinoma [238]. On the other hand, miR-331-3p is reported to play an anti-cancer role in prostate cancer by targeting the RALA pathway [239] and ERBB-2 mediated androgen receptor signaling [240], colorectal cancer by targeting HER2 [241], NSCLC by targeting ErbB2, VAV2 and inhibiting epithelial to mesenchymal transition [242], glioblastoma and cervical cancer by regulating NRP-2 [243,244], ovarian cancer by targeting RCC2 [245], urothelial cancer by targeting NACC1 [246], and gastric cancer by targeting E2F1 [247]. miR-331-3p also plays a role in inhibiting intracranial aneurysm by regulating TNF-α and CD14, as well as by maintaining contractile vascular smooth muscle [248].

The remaining of the 14 most important RPs regulating miRNAs are miR-92b-3p, miR-652-3p, miR-766-3p and miR-744-5p, respectively. Interestingly, miR-92b-3p acts as a preventive molecule against several neural and cardiac diseases [280]; for example, it facilitates the growth of neurite, and healing of acute spinal cord injury by mediating the PTEN/AKT pathway [249]. Another example is cardiac hypertrophy, which is suppressed in mice by miR-92b-3p, potentially by targeting MEF2D [250] and HAND2 [251]. Furthermore, it can inhibit the pulmonary artery derived smooth muscle cells proliferation by targeting USP28 [281], as well as regulate the assembly of primordial follicles in the ovaries of neonatal mice by targeting TSC1 [282]. In cancer, miR-92b-3p have both anti- and pro-cancer roles in different types of cancers: it suppresses pancreatic cancer by targeting GABRA3 [252], but promotes several others such as colorectal cancer by inhibiting FBXW7 [253], esophageal squamous cell carcinoma by target KLF4 and DCS2 [254], gastric cancer by downregulating MMP2, MMP9 and HOXD10 [255], and also could be a biomarker for synovial sarcoma [256]. Similarly, miR-652-3p is also reported to have both anti- and pro-cancer characteristics, it sensitizes lymphoblastic leukemia cells to chemotherapy [257] but promotes bladder cancer by targeting KCNN3 [258], NSCLC by targeting Lgl1 [259] as well as prostate cancer [260]. In addition, miR-652-3p inhibits healing of endothelial damage and atherosclerosis by downregulating Cyclin D2 [261] but promotes trophoblast cells proliferation potentially by targeting HOXA9 and regulating EphB4 [283]. miR-766-3p is also known as a dual player in cancer, it showed an anti-cancer effect on HCC by targeting WNT3A [262], but it is also known to inhibit cell-cycle progression and metastasis of RCC by targeting SF2 [263] and HCC by targeting MTA3 [284], respectively. Furthermore, it plays roles in anti-inflammatory signaling by indirectly inhibiting NF-κB signaling [285]. However, miR-744-5p is reported by only two publications and both claimed its inhibitory role in cancer, which includes ovarian cancer cells by targeting HNRNPC and NFIX [264] and NSCLC by targeting PAX2 [265]. Taken together, the 14 most important miRNAs based on their connection to the RPs (Figure 3b and Table 4) are well involved in different diseases, particularly in different types of cancers. However, their role in the regulation of RPs coding mRNAs is not really investigated, which is very important for understanding the impact of miRNAs on the functioning of translation machinery as well as the global synthesis of proteins.

## 8. Conclusions

I. The role of miRNAs in the regulation of gene expression is a well-investigated area of research, however, the roles of miRNAs in the regulation of RPs coding gene expression remains unexplored, and therefore, this area is required to be investigated further for a proper understanding of RPs synthesis, ribosomal assembly and regulation of global protein translation.
II. The idea of master miRNAs that can influence the global translation of proteins is potentially true, and the existence of such miRNAs is further assured by the report of Orom et al. 2008, which showed that miR-10a binds to the 5′UTR of the RPs and regulates the global protein synthesis. However, further investigations are required to establish it as a scientific fact.
III. RPs are an integral part of the translation machinery, required for the proper assembly and functioning of the ribosomes. Therefore, the ultimate results of the miRNA mediated regulation of the RPs are improper functioning of the translation machinery and deregulated synthesis proteins.
IV. Ribosomopathy refers to a group of diseases caused by the deformed translation machinery, and many RPs are directly involved with ribosomopathy, thus finding the regulatory interaction of miRNAs and RPs could explore the regulatory role of miRNAs on ribosomopathy and might help to develop future therapeutic strategies.
V. Deregulation of both RPs and miRNAs is very common in diseases including almost all types of cancers. Investigation of the miRNAs mediated regulation of RPs could provide a reasonable explanation behind the pathological conditions of these diseases.

## Figures and Tables

**Table 1:** Clustering of the human Ribosomal proteins (RPs) according to their involvement in the biological processes.

Biological Processes	Ribosomal Proteins (RPs) Involved
1. SRP-dependent cotranslational protein targeting to membrane (GO:0006614)	RPL4, RPL5, RPL30, RPL3, RPL32, RPL31, RPL34, RPLP1, RPLP0, RPL10A, RPL8, RPL9, RPL6, RPL7, RPS4X, RPS15, RPS14, RPL7A, RPS17, RPS16, RPS19, RPL18A, RPS18, RPL36, RPLP2, RPL35, RPL37, RPS11, RPL39, RPS10, RPS13, RPS12, RPS9, RPL21, RPS7, RPS8, RPL23, RPS5, RPL22, RPS6, RPL13A, RPS3A, RPSA, RPL24, RPL27, RPL26, UBA52, RPL10, RPL12, RPL36A, RPS4Y1, RPS15A, RPS3, RPL14, RPS2, RPL15, RPS27A, RPL18, RPL17, RPL19, RPL41, RPL23A, RPS26, RPS25, RPS28, RPS27, RPS29, RPL27A, RPS20, FAU, RPS21, RPS24, RPS23
2. Viral transcription (GO:0019083)	
3. Nuclear-transcribed mRNA catabolic process, nonsense-mediated decay (GO:0000184)	
4. Translational initiation (GO:0006413)	
5. rRNA processing (GO:0006364)	
6. Translation (GO:0006412)	
7. Cytoplasmic translation (GO:0002181)	RPL31, RPLP1, RPL22, RPLP0, RPL36A, RPL8, RPL9, RPL6, RPL7, RPL36, RPLP2, RPL26, RPL15
8. Ribosomal small subunit assembly (GO:0000028)	RPS15, RPS14, RPS17, RPS28, RPS27, RPS19, RPS5, RPSA, RPS10
9. Ribosomal small subunit biogenesis (GO:0042274)	RPS15, RPS17, RPS28, RPS16, RPS7, RPS19, RPS6, RPS24
10. Ribosomal large subunit assembly (GO:0000027)	RPL5, RPL3, RPL10, RPL12, RPL24, RPL23A, RPL6
11. Maturation of SSU-rRNA from tricistronic rRNA transcript (SSU-rRNA, 5.8S rRNA, LSU-rRNA) (GO:0000462)	RPS14, RPS16, RPS19, RPS8, RPS24
12. Ribosome biogenesis (GO:0042254)	RPL7A, RPS28, RPS18, RPL34, RPLP0
13. Cell-cell adhesion (GO:0098609)	RPS26, RPL7A, RPL34, RPL14, RPL24, RPL23A, RPL15, RPS2, RPL6
14. Ribosomal large subunit biogenesis (GO:0042273)	RPL5, RPL14, RPL26, RPL7
15. Liver regeneration (GO:0097421)	RPS16, RPL32, RPS24, RPL19
16. Maturation of SSU-rRNA (GO:0030490)	RPS14, RPS28, RPS19
17. Endonucleolytic cleavage to generate mature 3′-end of SSU-rRNA from (SSU-rRNA, 5.8S rRNA, LSU-rRNA) (GO:0000461)	RPSA, RPS21
18. DNA damage response, detection of DNA damage (GO:0042769)	RPS3, RPS27A, UBA52
19. Negative regulation of RNA splicing (GO:0033119)	RPS26, RPS13
20. Endonucleolytic cleavage in ITS1 to separate SSU-rRNA from 5.8S rRNA and LSU-rRNA from tricistronic rRNA transcript (SSU-rRNA, 5.8S rRNA, LSU-rRNA) (GO:0000447)	RPSA, RPS21
21. Maturation of LSU-rRNA from tricistronic rRNA transcript (SSU-rRNA, 5.8S rRNA, LSU-rRNA) (GO:0000463)	RPL35, RPL7
22. Erythrocyte homeostasis (GO:0034101)	RPS17, RPS24
23. Regulation of necrotic cell death (GO:0010939)	RPS27A, UBA52
24. Virion assembly (GO:0019068)	RPS27A, UBA52
25. Regulation of type I interferon production (GO:0032479)	RPS27A, UBA52
26. MyD88-independent toll-like receptor signaling pathway (GO:0002756)	RPS27A, UBA52
27. Maturation of LSU-rRNA (GO:0000470)	RPL7A, RPL10A
28. Translational elongation (GO:0006414)	RPLP1, RPLP2
29. Response to ethanol (GO:0045471)	RPS4X, RPL15, RPL10A
30. Error-free translesion synthesis (GO:0070987)	RPS27A, UBA52
31. Error-prone translesion synthesis (GO:0042276)	RPS27A, UBA52
32. Stress-activated MAPK cascade (GO:0051403)	RPS27A, UBA52
33. Notch signaling pathway (GO:0007219)	RPS19, RPS27A, UBA52
34. Nucleotide-excision repair, DNA duplex unwinding (GO:0000717)	RPS27A, UBA52
35. Nucleotide-excision repair, DNA damage recognition (GO:0000715)	RPS27A, UBA52
36. Positive regulation of epidermal growth factor receptor signaling pathway (GO:0045742)	RPS27A, UBA52
37. Nucleotide-excision repair, DNA gap filling (GO:0006297)	RPS27A, UBA52
38. Cellular response to interleukin-4 (GO:0071353)	RPL3, RPLP0

**Table 2:** Ribosomal proteins on the biogenesis and assembly of ribosomes, and translation of proteins.

Ribosomal Proteins	Functions	References
RPL33	Regulates the processing of the 35S and 27S pre-rRNAs	[28]
RPS20	Regulates mRNA binding and subunits association, mutation impairs 70S subunit formation and mRNA binding to the 30S subunit	[30]
RPL16	Assembly of 60S subunits	[31,52]
RP59	Assembly of the 40S subunit	[31]
RPL1	Maintain the stability of 5S rRNA and assembly of 60S subunits	[32,33]
RPL9	Maturation of the small subunit	[34]
RPL23	Chaperone-assisted folding of proteins	[37]
RPL35	Recognition of peptide and insertion to the translocation channel	[38]
RPS12	Mutations at lysine-42 of S12, increase accuracy of translation	[41]
RPS4	Mutation reduces the accuracy of translation	[41,43]
RPS5	Mutation reduces the accuracy of translation	[41]
RPS9	Maintain the accuracy of translation	[42]
RPS28	Maintain the accuracy of translation	[43]
RPL39	Maintain the accuracy of translation	[61]
RPL3	Regulates the peptidyltransferase activity and mutation alter the fidelity of translation	[45]
RPL5	Regulates the peptidyltransferase by helping the anchor of peptidyl-tRNA to the P-site	[47]
RPL41	Optimizes peptidyltransferase activity by regulating the translocation	[46]
RPL24	Regulates the P-site binding and kinetics of the protein synthesis	[61]
RPL10	Regulates nuclear exporting by interacting and releasing cytoplasmic Nmd3p from 60S subunit	[48,49,50]
RPL12	Assembly of ribosomal stalk	[51]
RPS14	Maturation of 43S preribosomes	[53]
RPS0	20S rRNA-precursor to mature 18S rRNA	[54,55]
RPS21	Maturation of the 3′ end of 18S rRNA	[55]
RPL25	Pre-rRNA processing	[56]
RPS15	Regulates the nuclear exit of the 40S subunit precursors	[57]

**Table 3:** Involvement of ribosomal proteins in different diseases.

Major Ribosome Related Diseases	Gene Involved	Reference
Diamond–Blackfan anemia (DBA)	RPS19, RPS26, RPL5, RPL11	[70,71,72]
5q-syndrome	RPS14	[73]
Schwachman-Diamond syndrome (SDS)	SBDS	[74]
X-linked dyskeratosis congenita (DC)	DKC1	[75]
Cartilage hair hypoplasia (CHH)	RMRP	[76]
Treacher Collins syndrome (TCS)	TCOF1	[77]
Bowen–Conradi syndrome (BCS)	EMG1	[78,79,80]
North American Indian childhood cirrhosis (NAIC)	CIRH1A	[81,82]
Non-small cell lung cancer	RPS6, RPS15A	[83,84]
Ovarian cancer	RPS4X	[85]
Bladder cancer	RPS4X	[86]
Prostate cancer	RPL31	[87]
Esophageal cancer	RPL34	[88]
T-cell lymphoma	RPL22	[89]
Colorectal cancer	RPS20	[90]

**Table 4:** The top 14 miRNAs that are predicted to target most of the ribosomal proteins (RPs), along with their reported association to the diseases.

miRNA	Predicted Target Ribosomal Proteins	Reported Disease Association
hsa-mir-16-5p	RPSA, RPL4, RPL9, RPL30, RPS3A, RPS15A, RPS24, RPL12, RPL3, RPL31, RPS6, RPL10, RPL27A, RPLP2, RPS27, RPS5, RPL14, RPL36, RPL5, RPL6, RPL21, RPLP0, RPLP1, RPS2, RPS3, RPS12, RPS17, RPS25, RPS14, RPL19, RPL15, RPL35, RPS19, RPS11	breast cancer [147,148], hepatocellular carcinoma (HCC) [149], mesothelioma [150], glioma [151], neuroblastoma [152], chordoma, gastric cancer and osteoarthritis [153,154,155], osteoclastogenesis [156], and rheumatoid arthritis [157].
hsa-mir-92a-3p	RPL9, RPL30, RPS3A, RPS10, RPS15A, RPS24, RPL18A, RPL3, RPL27A, RPS5, RPLP1, RPS25, RPL37, RPL7A, RPL24, FAU, RPS14, RPS28, RPL23, RPL13A, RPL39, RPL22, RPS23, RPL8, RPL15, RPL27, RPS8, RPS15	melanoma [158], liposarcoma [159], glioma [160], leukemia [161], colorectal cancer [162,163], degradation of cartilage [164], Kawasaki disease [165], schizophrenia [166], systemic lupus erythematosus [167], and white matter impairment and post-stroke depression [168].
hsa-mir-100-5p	RPS15A, RPL12, RPL31, RPL10, RPS27, RPL14, RPL5, RPL21, RPLP1, RPS2, RPL7, RPL36A, RPL7A, RPL26, RPL19, RPL15, RPS8, RPS15, RPL10A	prostate cancer [169], RCC [170], oral cancer [171], NSCLC [172], HCC [173], abeta-induced pathologies [174], and hidradenitis suppurativa [175].
hsa-mir-615-3p	RPSA, RPL9, RPL3, UBA52, RPL31, RPL36, RPL5, RPL21, RPLP1, RPS2, RPS3, RPS12, RPS17, RPL7, RPL7A, RPL23, RPL15, RPS15	gastric cancer [176], prostate cancer [177], NSCLC [178,179], esophageal cancer [180], and HCC [181].
hsa-mir-484	RPSA, RPS10, RPS24, RPL3, UBA52, RPL27A, RPLP2, RPL36, RPL5, RPL21, RPLP0, RPLP1, RPS3, FAU, RPS14, RPL13A, RPS18, RPS23, RPS15, RPS29, RPS9, RPL23A	cervical cancer [182,183], gastric cancer [184], glioma [185], NSCLC [186], adrenocortical cancer [187], colorectal cancer [188], renal carcinoma [189], and breast cancer [190].
hsa-mir-186-5p	RPL4, RPL9, RPS4X, RPL18A, RPL3, RPL14, RPL36, RPL5, RPLP0, RPLP1, RPS2, RPS3, RPS7, RPS26, RPL15, RPL27, RPS29, RPL32, RPS21	ischemia stroke [191], hippocampal neurons [192], acute coronary syndrome [193], reproductive health [194], osteosarcoma [195,196], colorectal cancer [197], NSCLC and neuroblastoma [198], lung adenocarcinoma [199], and prostate cancer [200].
hsa-mir-320a	RPL9, RPL30, RPS4X, UBA52, RPL10, RPS27, RPL36, RPLP1, RPS2, RPS12, RPS17, RPS16, RPL7A, RPL13A, RPL8, RPL15, RPL27	HCC [201,202], NSCLC [203], gliomas [204], gastric cancer [205,206], lung adenocarcinoma [207], tongue squamous cell carcinoma [208], multiple myeloma [209], breast cancer [210], colorectal cancer [211], bladder carcinoma [212], diabetic nephropathy [213], cartilage degradation [214], osteoporosis [215,216], cardiotoxicity [217], anomalous placentation [218], atherogenesis [219], arrhythmogenic cardiomyopathy [220], and polycystic ovary syndrome [221].
hsa-mir-193b-3p	RPL9, RPS10, RPL12, RPS6, RPL27A, RPL6, RPLP0, RPS3, FAU, RPL26, RPS18, RPL22, RPL8, RPS21, RPL23A	preeclampsia [222], ovarian cancer [223], breast cancer [224], and urothelial cancer [225].
hsa-let-7a-5p	RPSA, RPL4, RPL9, RPL30, RPS3A, RPS4X, RPS10, RPS13, RPS15A, RPS24, RPL31, RPS27, RPLP1, RPS14, RPL8, RPS29	osteogenesis [226], lung cancer [227,228], HCC [229], colorectal cancer [230], leukemia cells [231], diabetic nephropathy [232], hepatic fibrosis [233].
hsa-mir-331-3p	RPS4Y1, RPS27, RPLP0, RPLP1, RPS2, RPS3, RPS12, RPL36A, RPL7A, RPS14, RPL13A, RPS29, RPS9, RPL34	pancreatic cancer [234], HCC [235,236,237], esophageal adenocarcinoma [238], prostate cancer [239,240], colorectal cancer [241], NSCLC [242], glioblastoma and cervical cancer [243,244], ovarian cancer [245], urothelial cancer [246], and gastric cancer [247], intracranial aneurysm [248].
hsa-mir-92b-3p	RPL9, RPL30, RPS3A, RPS4X, RPL3, RPLP0, RPL37, RPL24, RPS14, RPS28, RPL23, RPL8	acute spinal cord injury [249], cardiac hypertrophy [250,251], pancreatic cancer [252], colorectal cancer [253], esophageal squamous cell carcinoma [254], gastric cancer [255], and synovial sarcoma [256].
hsa-mir-652-3p	RPL4, RPL18, RPL18A, RPS6, RPL21, RPLP1, RPS12, RPS16, RPL26, RPL19, RPS23, RPL27, RPS29, RPL32, RPS19	lymphoblastic leukemia [257], bladder cancer [258], NSCLC [259], prostate cancer [260], and atherosclerosis [261].
hsa-mir-766-3p	RPS15A, RPL12, UBA52, RPL27A, RPLP2, RPL23, RPS26, RPL27, RPS9, RPS19, RPS21	HCC [262], and RCC [263].
hsa-mir-744-5p	RPL4, RPL18, RPL18A, RPL3, RPS6, RPL10, RPL36, RPLP1, RPL37, RPL7A, RPS14	ovarian cancer [264], and NSCLC [265].

## Supplementary Materials

The following are available online at https://www.mdpi.com/2073-4409/10/1/110/s1, Supplementary Figure S1. The RNA-seq heatmap showing the expression status of RPs in 300 ovarian cancer patients, Supplementary Figure S2. Interaction network between ribosomal proteins (RPs) and microRNAs (miRNAs). The extensive network is showing that more than a thousand miRNAs are connected to the 72 RPs, Supplementary Table S1. Involvement of Ribosomal Proteins (RPs) in different cellular components, Supplementary Table S2. Involvement of Ribosomal Proteins (RPs) in different Molecular Functions, and Supplementary Table S3. Top 15 ribosomal proteins (RP) in terms of being targeted by the numbers of miRNAs.
