# Supplementary material for: microRNAs Mediated Regulation of the Ribosomal Proteins and its Consequences on the Global Translation of Proteins
Source: Cells. 2021 Jan 8;10(1):110. doi: 10.3390/cells10010110 (PMC7827472; doi:10.3390/cells10010110)
Supplement: Supplementary file 1 [file cells-10-00110-s001.pdf]

Supplementary Data

Review

microRNAs Mediated Regulation of the Ribosomal Proteins and its Consequences on the Global Translation of Proteins

Abu Musa Md Talimur Reza [1] and Yu-Guo Yuan [2,3,*]

[1] Institute of Biochemistry and Biophysics, Polish Academy of Sciences, Pawińskiego 5a, 02-106 Warsaw, Poland; talimurku@gmail.com
[2] College of Veterinary Medicine/Jiangsu Co-innovation Center for Prevention and Control of Important Animal Infectious Diseases and Zoonoses, Yangzhou University, Yangzhou 225009, China
[3] Jiangsu Key Laboratory of Zoonosis/Joint International Research Laboratory of Agriculture and Agri-Product Safety, the Ministry of Education of China, Yangzhou University, Yangzhou 225009, China
* Correspondence: ygyuan@yzu.edu.cn; Tel.: +86-514-8797-9228

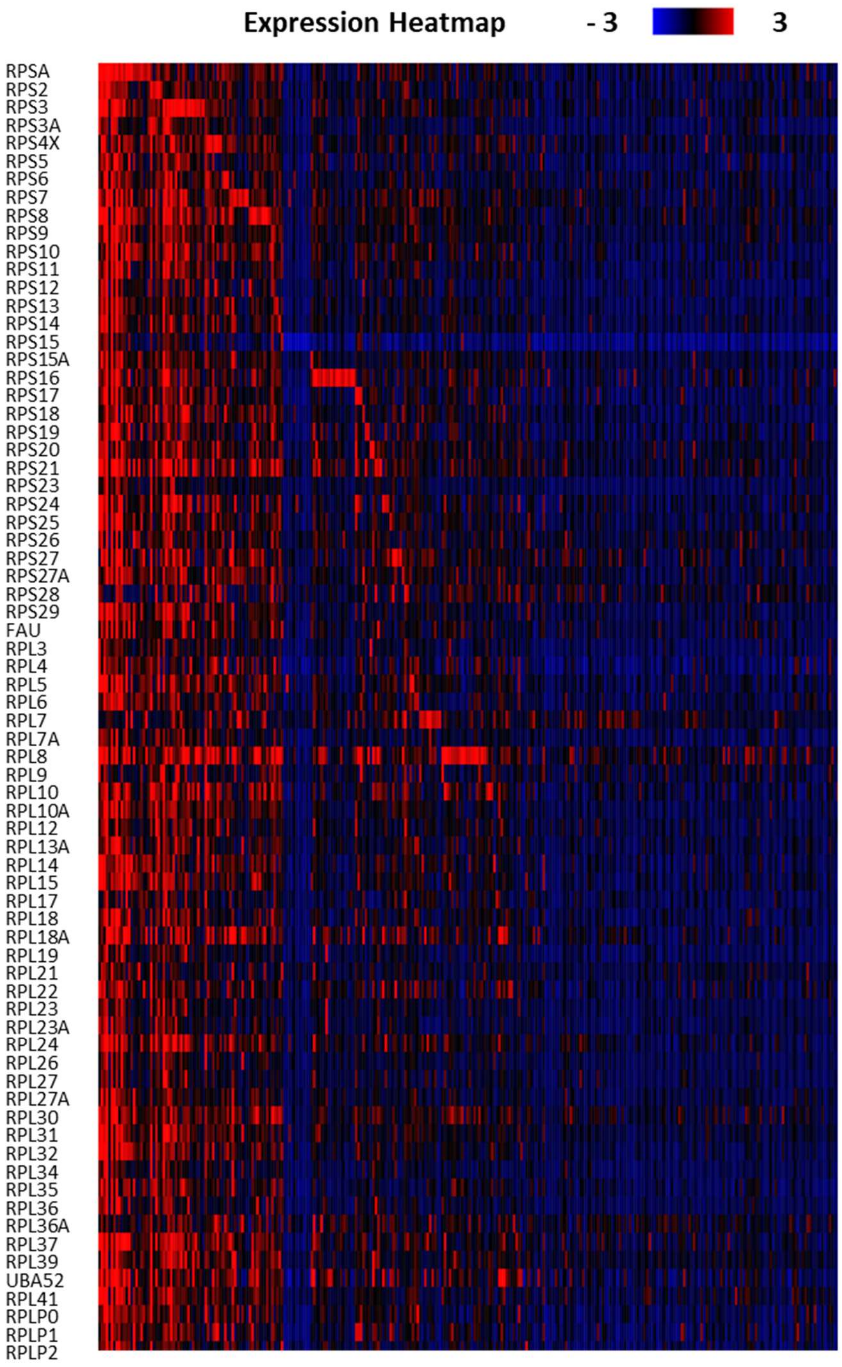

Supplementary Figure S1. The RNA-seq heatmap showing the expression status of RPs in 300 ovarian cancer patients. The data was generated using the cBioPortal for Cancer Genomics (http://www.cbioportal.org/index.do) database from the cancer genome atlas (TCGA) Research Network (https://www.cancer.gov/about-nci/organization/ccg/research/structural-genomics/tcga).

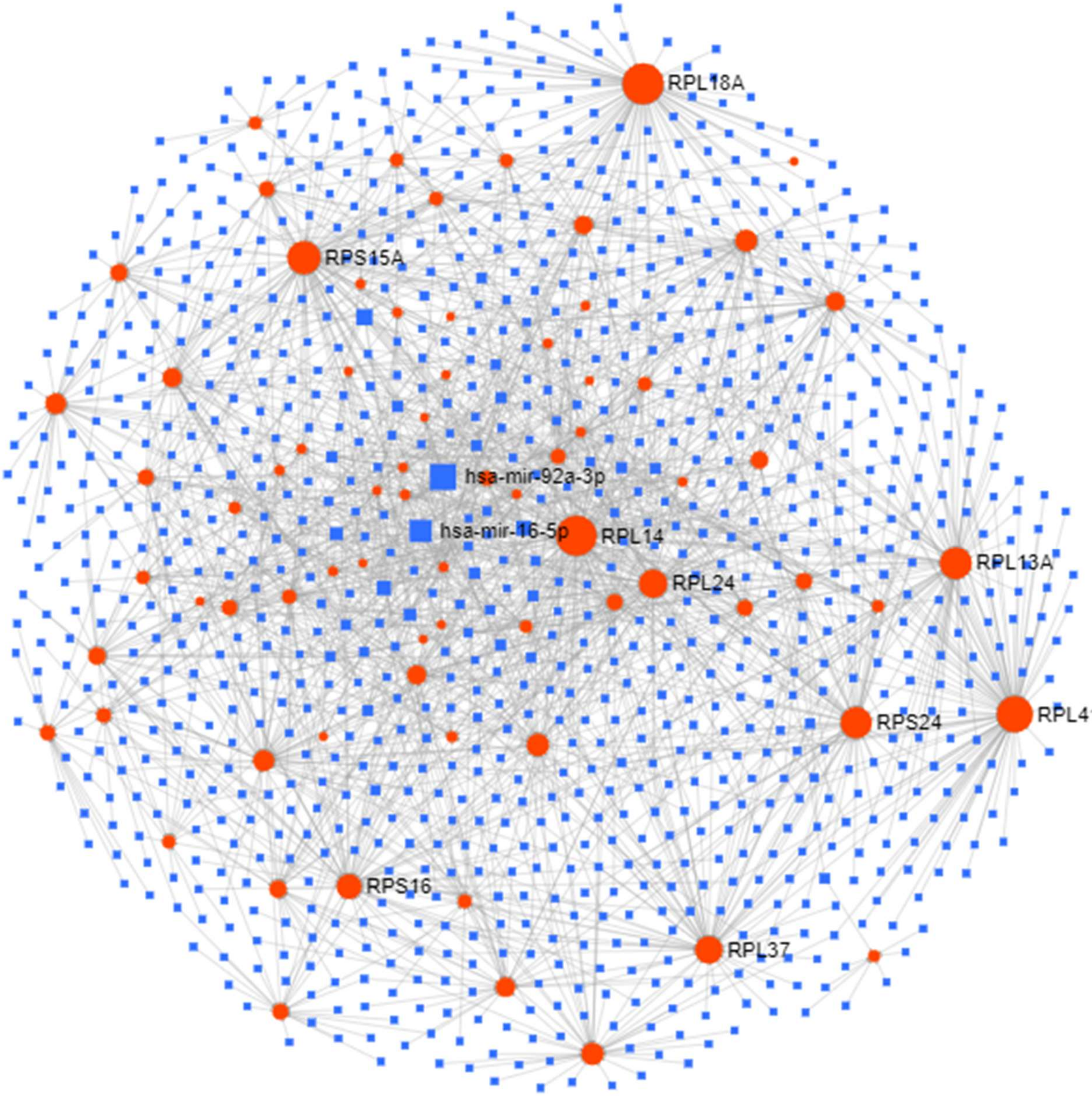

Supplementary Figure S2. Interaction network between ribosomal proteins (RPs) and microRNAs (miRNAs). The extensive network is showing that more than a thousand miRNAs are connected to the 72 RPs. The size of the nodes indicates the degree of connectivity, bigger the node size higher the connection. The interaction network was generated using miRNet database (https://www.mirnet.ca/).

Supplementary Table S1. Involvement of Ribosomal Proteins (RPs) in different cellular components

**Cellular Component**	**Ribosomal Proteins (RPs) Involved**
GO:0005840~ribosome	RPL4, RPL5, RPL30, RPL3, RPL32, RPL31, RPL34, RPLP1, RPLP0, RPL10A, RPL8, RPL9, RPL6, RPL7, RPS4X, RPS15, RPS14, RPL7A, RPS17, RPS16, RPS19, RPL18A, RPS18, RPL36, RPLP2, RPL35, RPL37, RPS11, RPS10, RPS13, RPS9, RPL21, RPS7, RPS8, RPL23, RPL22, RPS6, RPL13A, RPS3A, RPL24, RPL27, UBA52, RPL10, RPL36A, RPS4Y1, RPS15A, RPS3, RPL14, RPS2, RPL15, RPS27A, RPL18, RPL19, RPL41, RPL23A, RPS26, RPS25, RPS28, RPS27, RPS29, RPL27A, RPS20, FAU, RPS21, RPS24, RPS23
GO:0022625~cytosolic large ribosomal subunit	RPL4, RPL5, RPL30, RPL3, RPL32, RPL10, RPL31, RPL34, RPLP1, RPL12, RPLP0, RPL36A, RPL10A, RPL8, RPL9, RPL6, RPL7, RPL7A, RPL18A, RPL36, RPL14, RPL35, RPLP2, RPL37, RPL15, RPL18, RPL39, RPL17, RPL19, RPL41, RPL21, RPL23, RPL22, RPL13A, RPL23A, RPL27A, RPL24, RPL27, RPL26, UBA52
GO:0022627~cytosolic small ribosomal subunit	RPS4Y1, RPS15, RPS4X, RPS14, RPS17, RPS15A, RPS16, RPS19, RPS18, RPS3, RPS2, RPS27A, RPS11, RPS10, RPS13, RPS12, RPS9, RPS7, RPS8, RPS5, RPS6, RPSA, RPS3A, RPS26, RPS25, RPS28, RPS27, RPS29, RPS20, FAU, RPS21, RPS24, RPS23
GO:0005829~cytosol	RPL4, RPL5, RPL30, RPL3, RPL32, RPL31, RPL34, RPLP1, RPLP0, RPL10A, RPL8, RPL9, RPL6, RPL7, RPS4X, RPS15, RPS14, RPL7A, RPS17, RPS16, RPS19, RPL18A, RPS18, RPL36, RPLP2, RPL35, RPL37, RPS11, RPL39, RPS10, RPS13, RPS12, RPS9, RPL21, RPS7, RPS8, RPL23, RPS5, RPL22, RPS6, RPL13A, RPS3A, RPSA, RPL24, RPL27, RPL26, UBA52, RPL10, RPL12, RPL36A, RPS4Y1, RPS15A, RPS3, RPL14, RPS2, RPL15, RPS27A, RPL18, RPL17, RPL19, RPL41, RPL23A, RPS26, RPS25, RPS28, RPS27, RPS29, RPL27A, RPS20, FAU, RPS21, RPS24, RPS23
GO:0005925~focal adhesion	RPL4, RPL5, RPL30, RPL3, RPL31, RPLP1, RPL12, RPLP0, RPL10A, RPL8, RPL9, RPL6, RPL7, RPS15, RPS4X, RPS14, RPL7A, RPS17, RPS16, RPS19, RPS18, RPS3, RPLP2, RPS2, RPS11, RPL18, RPS10, RPS13, RPL19, RPS9, RPS7, RPS8, RPL23, RPS5, RPL22, RPL13A, RPS3A, RPS29, RPL27
GO:0015935~small ribosomal subunit	RPS9, RPS5, RPS6, RPSA, RPS15, RPS4X, RPS26, RPS25, RPS28, RPS16, RPS18, RPS29, RPS3, RPS20, RPS2, FAU, RPS27A, RPS21, RPS24, RPS23
GO:0016020~membrane	RPL4, RPL5, RPL30, RPL32, RPL31, RPLP0, RPL10A, RPL8, RPL9, RPL6, RPL7, RPS4X, RPS15, RPS14, RPL7A, RPS17, RPS16, RPS19, RPL18A, RPS18, RPL36, RPLP2, RPL35, RPS11, RPS10, RPS13, RPS12, RPS9, RPL21, RPS7, RPS8, RPL23, RPS5, RPS6, RPL13A, RPSA, RPL24, RPL27, RPL26, RPL10, RPL12, RPS4Y1, RPS15A, RPS3, RPL14, RPS2, RPL15, RPS27A, RPL18, RPL19, RPS26, RPL27A, RPS20, RPS24, RPS23
GO:0005730~nucleolus	RPL4, RPL5, RPL3, RPL34, RPL12, RPL10A, RPL8, RPL9, RPL7, RPS14, RPL7A, RPS19, RPL36, RPS3, RPL35, RPS2, RPS27A, RPS11, RPL18, RPS10, RPS13, RPL19, RPS9, RPL21, RPS7, RPL23, RPS6, RPL13A, RPL23A, RPS3A, RPS26, RPS25, RPS23
GO:0031012~extracellular matrix	RPL30, RPS7, RPL23, RPS5, RPL12, RPL22, RPS3A, RPL9, RPS4X, RPS14, RPS25, RPS17, RPS15A, RPS16, RPS19, RPS18, RPS3, RPL27, RPS20, RPS11, RPS10, RPS13
GO:0070062~extracellular exosome	RPL4, RPL5, RPL30, RPL3, RPL31, RPL34, RPLP1, RPL12, RPLP0, RPL10A, RPL7, RPS4X, RPS14, RPL7A, RPS17, RPS15A, RPS16, RPS19, RPS18, RPL14, RPS3, RPLP2, RPS2, RPL15, RPS27A, RPS11, RPS10, RPS13, RPS9, RPS7, RPS8, RPL23, RPS5, RPL22, RPL23A, RPSA, RPS3A, RPS26, RPS25, RPS28, RPS29, RPL24, RPL27, RPS20, RPL26, UBA52
GO:0030529~intracellular ribonucleoprotein complex	RPL4, RPL5, RPS9, RPS7, RPS8, RPS5, RPL22, RPS6, RPLP0, RPL13A, RPS3A, RPL7, RPS4X, RPS3, RPL27

GO:0005654~nucleoplasm	RPL3, RPS4Y1, RPS15, RPS4X, RPS14, RPS17, RPS15A, RPS16, RPS19, RPS18, RPS3, RPS2, RPS27A, RPS11, RPS10, RPS13, RPS12, RPS9, RPS7, RPS8, RPS5, RPS6, RPSA, RPS3A, RPS26, RPS25, RPS28, RPS27, RPS29, RPS20, FAU, UBA52, RPS21, RPS24, RPS23
GO:0005737~cytoplasm	RPL4, RPL5, RPL30, RPL3, RPL34, RPLP1, RPLP0, RPL10A, RPL8, RPL9, RPL7, RPS15, RPS14, RPL7A, RPS15A, RPS19, RPL36, RPL14, RPS3, RPL35, RPS2, RPS27A, RPS11, RPL18, RPL19, RPS9, RPL21, RPS7, RPS8, RPL23, RPL22, RPS6, RPL13A, RPL23A, RPSA, RPS3A, RPS26, RPS25, RPS28, RPS29, RPL24, RPS20, RPS21, RPS24, RPS23
GO:0015934~large ribosomal subunit	RPL27A, RPL13A, RPL26, RPL10A, RPL17
GO:0005913~cell-cell adherens junction	RPS26, RPL7A, RPL34, RPL14, RPL24, RPL23A, RPL15, RPS2, RPL6
GO:0036464~cytoplasmic ribonucleoprotein granule	RPS4X, RPS6, RPLP0, RPL6
GO:0005844~polysome	RPS4X, RPS6, RPS3, RPS4Y1
GO:0005634~nucleus	RPL4, RPL5, RPL30, RPL3, RPLP0, RPL10A, RPS4Y1, RPL9, RPL6, RPL7, RPS15, RPL7A, RPS18, RPS3, RPS2, RPL15, RPS27A, RPL18, RPL17, RPS13, RPS9, RPS7, RPS8, RPL22, RPS6, RPL13A, RPL23A, RPSA, RPS3A, RPS25, RPS27, RPL27, UBA52, RPS24
GO:0030687~preribosome, large subunit precursor	RPLP1, RPLP0, RPLP2
GO:0030686~90S preribosome	RPS7, RPSA

Supplementary Table S2. Involvement of Ribosomal Proteins (RPs) in different Molecular Functions

Molecular Functions	Ribosomal Proteins (RPs) Involved
GO:0003735~structural constituent of ribosome	RPL4, RPL5, RPL30, RPL3, RPL32, RPL31, RPL34, RPLP1, RPLP0, RPL10A, RPL8, RPL9, RPL6, RPL7, RPS4X, RPS15, RPS14, RPL7A, RPS17, RPS16, RPS19, RPL18A, RPS18, RPL36, RPLP2, RPL35, RPL37, RPS11, RPL39, RPS10, RPS13, RPS12, RPS9, RPL21, RPS7, RPS8, RPL23, RPS5, RPL22, RPS6, RPL13A, RPS3A, RPSA, RPL24, RPL27, RPL26, UBA52, RPL10, RPL12, RPL36A, RPS4Y1, RPS15A, RPS3, RPL14, RPS2, RPL15, RPS27A, RPL18, RPL17, RPL19, RPL41, RPL23A, RPS26, RPS28, RPS27, RPS29, RPL27A, RPS20, FAU, RPS21, RPS24, RPS23
GO:0044822~poly(A) RNA binding	RPL4, RPL5, RPL30, RPL3, RPL32, RPL31, RPLP0, RPL10A, RPL8, RPL6, RPL7, RPS4X, RPS15, RPS14, RPL7A, RPS17, RPS16, RPS19, RPL18A, RPS18, RPL36, RPL35, RPS11, RPS10, RPS13, RPS12, RPS9, RPL21, RPS7, RPS8, RPL23, RPS5, RPL22, RPS6, RPL13A, RPS3A, RPSA, RPL24, RPL27, RPL26, RPL10, RPL12, RPL36A, RPS15A, RPS3, RPL14, RPS2, RPL15, RPS27A, RPL17, RPL19, RPL23A, RPS26, RPS25, RPS28, RPS27, RPL27A, RPS20, FAU, RPS21, RPS24, RPS23
GO:0003723~RNA binding	RPL4, RPL5, RPL30, RPL3, RPL31, RPL34, RPL10A, RPS4Y1, RPL8, RPL9, RPL6, RPL7, RPS15, RPS4X, RPS14, RPL7A, RPS15A, RPS16, RPL18A, RPS18, RPL14, RPS3, RPL37, RPS2, RPL15, RPL18, RPL39, RPL19, RPL41, RPL21, RPS7, RPS5, RPL22, RPS3A, RPS25, RPS28, RPL27A, RPL24, RPS20, RPL26, FAU
GO:0019843~rRNA binding	RPS4X, RPS9, RPS18, RPS5, RPL12, RPL23A, RPL37, RPS4Y1, RPL8, RPS11, RPL9
GO:0070180~large ribosomal subunit rRNA binding	RPLP1, RPL23, RPLP0, RPLP2, RPL19
GO:0003729~mRNA binding	RPS26, RPS5, RPS3, RPL13A, RPL35, RPS2, RPS13, RPL7
GO:0098641~cadherin binding involved in cell-cell adhesion	RPS26, RPL7A, RPL34, RPL14, RPL24, RPL23A, RPL15, RPS2, RPL6
GO:1990932~5.8S rRNA binding	RPL8, RPS13, RPL19
GO:0005515~protein binding	RPL4, RPL5, RPL30, RPL3, RPL31, RPLP1, RPLP0, RPL10A, RPL9, RPL7, RPS4X, RPS15, RPS14, RPL7A, RPS16, RPS19, RPL18A, RPS18, RPLP2, RPS11, RPS10, RPS13, RPS9, RPL21, RPS7, RPL23, RPS5, RPL22, RPS6, RPS3A, RPSA, RPL24, RPL26, UBA52, RPL10, RPL12, RPL36A, RPS15A, RPS3, RPL14, RPS2, RPL15, RPS27A, RPL17, RPL19, RPL23A, RPS26, RPS25, RPS28, RPS27, RPL27A, RPS20, RPS23
GO:0070181~small ribosomal subunit rRNA binding	RPS14, RPS3, RPS13
GO:0008097~5S rRNA binding	RPL5, RPL3
GO:0045182~translation regulator activity	RPS14, RPS9
GO:0017134~fibroblast growth factor binding	RPS19, RPS2

Supplementary Table S3. Top 15 ribosomal proteins (RP) in terms of being targeted by the numbers of miRNAs

**Ribosomal Protein**	**miRNA**
RPL41	hsa-mir-24-3p, hsa-mir-198, hsa-mir-222-3p, hsa-mir-23b-3p, hsa-mir-124-3p, hsa-mir-383-5p, hsa-mir-324-5p, hsa-mir-338-3p, hsa-mir-323b-5p, hsa-mir-519c-5p, hsa-mir-518f-5p, hsa-mir-526a, hsa-mir-517-5p, hsa-mir-513a-5p, hsa-mir-544a, hsa-mir-556-5p, hsa-mir-584-5p, hsa-mir-624-5p, hsa-mir-634, hsa-mir-767-3p, hsa-mir-148a-5p, hsa-mir-218-2-3p, hsa-mir-188-3p, hsa-mir-193a-5p, hsa-mir-455-3p, hsa-mir-760, hsa-mir-523-5p, hsa-mir-518e-5p, hsa-mir-522-5p, hsa-mir-519a-5p, hsa-mir-519b-5p, hsa-mir-520c-5p, hsa-mir-518d-5p, hsa-mir-663b, hsa-mir-1256, hsa-mir-1914-5p, hsa-mir-3128, hsa-mir-3168, hsa-mir-4305, hsa-mir-4257, hsa-mir-4253, hsa-mir-2355-5p, hsa-mir-4268, hsa-mir-4285, hsa-mir-4330, hsa-mir-3672, hsa-mir-3945, hsa-mir-4448, hsa-mir-4461, hsa-mir-4524a-3p, hsa-mir-4645-3p, hsa-mir-4670-3p, hsa-mir-4685-5p, hsa-mir-4716-5p, hsa-mir-4719, hsa-mir-4720-5p, hsa-mir-4751, hsa-mir-4757-5p, hsa-mir-4761-5p, hsa-mir-4777-5p, hsa-mir-4436b-3p, hsa-mir-2467-3p, hsa-mir-4795-3p, hsa-mir-4799-3p, hsa-mir-4433a-5p, hsa-mir-4524b-3p, hsa-mir-5581-3p, hsa-mir-5588-5p, hsa-mir-5588-3p, hsa-mir-5690, hsa-mir-758-5p, hsa-mir-4632-5p, hsa-mir-6071, hsa-mir-6077, hsa-mir-6502-5p, hsa-mir-6717-5p, hsa-mir-410-5p, hsa-mir-494-5p, hsa-mir-1301-5p, hsa-mir-513b-3p, hsa-mir-6729-3p, hsa-mir-6735-5p, hsa-mir-6738-3p, hsa-mir-6744-3p, hsa-mir-6769a-5p, hsa-mir-6772-3p, hsa-mir-6796-3p, hsa-mir-6828-3p, hsa-mir-6837-5p, hsa-mir-6847-5p, hsa-mir-6769b-5p, hsa-mir-6862-5p, hsa-mir-6864-3p, hsa-mir-6868-5p, hsa-mir-6878-3p, hsa-mir-6879-5p, hsa-mir-7108-5p, hsa-mir-7113-5p, hsa-mir-7843-5p, hsa-mir-7854-3p, hsa-mir-8076, hsa-mir-1-3p, hsa-mir-873-5p
RPL14	hsa-let-7b-5p, hsa-mir-15a-5p, hsa-mir-16-5p, hsa-mir-17-5p, hsa-mir-20a-5p, hsa-mir-93-5p, hsa-mir-100-5p, hsa-mir-106a-5p, hsa-mir-197-3p, hsa-mir-129-5p, hsa-mir-181a-5p, hsa-mir-15b-5p, hsa-mir-122-5p, hsa-mir-124-3p, hsa-mir-150-5p, hsa-mir-186-5p, hsa-mir-195-5p, hsa-mir-155-5p, hsa-mir-106b-5p, hsa-mir-302a-3p, hsa-mir-302b-3p, hsa-mir-302c-3p, hsa-mir-302d-3p, hsa-mir-372-3p, hsa-mir-373-3p, hsa-mir-424-5p, hsa-mir-20b-5p, hsa-mir-497-5p, hsa-mir-512-3p, hsa-mir-520e, hsa-mir-520f-3p, hsa-mir-520a-3p, hsa-mir-526b-3p, hsa-mir-520b, hsa-mir-520c-3p, hsa-mir-519d-3p, hsa-mir-520d-3p, hsa-mir-520g-3p, hsa-mir-520h, hsa-mir-566, hsa-mir-588, hsa-mir-612, hsa-mir-624-5p, hsa-mir-654-5p, hsa-mir-769-5p, hsa-mir-92a-2-5p, hsa-mir-186-3p, hsa-mir-323a-5p, hsa-mir-20b-3p, hsa-mir-541-3p, hsa-mir-876-3p, hsa-mir-1285-3p, hsa-mir-302e, hsa-mir-2114-5p, hsa-mir-2116-3p, hsa-mir-548s, hsa-mir-3176, hsa-mir-3178, hsa-mir-3187-3p, hsa-mir-4253, hsa-mir-3622b-5p, hsa-mir-3663-5p, hsa-mir-3910, hsa-mir-3945, hsa-mir-4435, hsa-mir-4473, hsa-mir-4479, hsa-mir-4484, hsa-mir-548an, hsa-mir-3187-5p, hsa-mir-3960, hsa-mir-4635, hsa-mir-4701-5p, hsa-mir-4731-5p, hsa-mir-4755-3p, hsa-mir-4763-5p, hsa-mir-5089-5p, hsa-mir-5189-5p, hsa-mir-5588-3p, hsa-mir-5589-5p, hsa-mir-6131, hsa-mir-6506-5p, hsa-mir-6720-3p, hsa-mir-619-5p, hsa-mir-6747-3p, hsa-mir-6768-3p, hsa-mir-6769a-5p, hsa-mir-6778-3p, hsa-mir-6838-5p, hsa-let-7b-5p, hsa-mir-15a-5p, hsa-mir-16-5p, hsa-mir-17-5p, hsa-mir-20a-5p, hsa-mir-93-5p, hsa-mir-100-5p, hsa-mir-106a-5p, hsa-mir-197-3p, hsa-mir-129-5p, hsa-mir-181a-5p, hsa-mir-15b-5p, hsa-mir-122-5p, hsa-mir-124-3p, hsa-mir-150-5p, hsa-mir-186-5p, hsa-mir-195-5p, hsa-mir-155-5p, hsa-mir-106b-5p, hsa-mir-302a-3p, hsa-mir-302b-3p, hsa-mir-302c-3p, hsa-mir-302d-3p, hsa-mir-372-3p, hsa-mir-373-3p, hsa-mir-424-5p, hsa-mir-20b-5p, hsa-mir-497-5p, hsa-mir-512-3p
RPL18A	hsa-let-7b-5p, hsa-let-7c-5p, hsa-mir-20a-5p, hsa-mir-92a-3p, hsa-mir-181b-5p, hsa-mir-191-5p, hsa-mir-149-5p, hsa-mir-186-5p, hsa-mir-106b-5p, hsa-mir-301a-3p, hsa-mir-378a-5p, hsa-mir-412-3p, hsa-mir-490-3p, hsa-mir-494-3p, hsa-mir-520a-5p, hsa-mir-525-5p, hsa-mir-503-5p, hsa-mir-18a-3p, hsa-mir-558, hsa-mir-650, hsa-mir-652-3p, hsa-mir-421, hsa-mir-769-3p, hsa-mir-22-5p, hsa-mir-30b-3p, hsa-mir-149-3p, hsa-mir-377-5p, hsa-mir-423-5p, hsa-mir-486-3p, hsa-mir-488-3p, hsa-mir-455-3p, hsa-mir-744-5p, hsa-mir-1227-3p, hsa-mir-1200, hsa-mir-1262, hsa-mir-1827, hsa-mir-544b, hsa-mir-3157-5p, hsa-mir-3160-3p, hsa-mir-3180-5p, hsa-mir-3192-5p, hsa-mir-4324, hsa-mir-4257, hsa-mir-4260, hsa-mir-4252, hsa-mir-4290, hsa-mir-4329, hsa-mir-3612, hsa-mir-3622b-5p, hsa-mir-3672, hsa-mir-

	3689a-3p, hsa-mir-3689b-3p, hsa-mir-3940-3p, hsa-mir-642b-3p, hsa-mir-4444, hsa-mir-3689c, hsa-mir-4487, hsa-mir-4682, hsa-mir-4701-3p, hsa-mir-4728-5p, hsa-mir-2467-3p, hsa-mir-642a-3p, hsa-mir-5002-5p, hsa-mir-5572, hsa-mir-873-3p, hsa-mir-1227-5p, hsa-mir-6086, hsa-mir-6499-3p, hsa-mir-6511a-5p, hsa-mir-210-5p, hsa-mir-1910-3p, hsa-mir-6736-5p, hsa-mir-6752-3p, hsa-mir-6769a-3p, hsa-mir-6779-5p, hsa-mir-6780a-5p, hsa-mir-6784-5p, hsa-mir-6785-5p, hsa-mir-6799-5p, hsa-mir-6801-3p, hsa-mir-6810-3p, hsa-mir-6856-3p, hsa-mir-6864-3p, hsa-mir-6883-5p, hsa-mir-6889-3p, hsa-mir-6892-3p, hsa-mir-7106-5p, hsa-mir-1273h-5p, hsa-mir-6516-5p, hsa-mir-7851-3p, hsa-mir-450a-2-3p, hsa-mir-8485, hsa-mir-1-3p
RPS15A	hsa-let-7a-5p, hsa-mir-16-5p, hsa-mir-17-5p, hsa-mir-23a-3p, hsa-mir-27a-3p, hsa-mir-29a-3p, hsa-mir-30a-5p, hsa-mir-92a-3p, hsa-mir-100-5p, hsa-mir-29b-3p, hsa-mir-30c-5p, hsa-mir-30d-5p, hsa-mir-7-5p, hsa-mir-10a-5p, hsa-mir-10b-5p, hsa-mir-34a-5p, hsa-mir-183-5p, hsa-mir-210-3p, hsa-mir-216a-5p, hsa-mir-23b-3p, hsa-mir-27b-3p, hsa-mir-122-5p, hsa-mir-124-3p, hsa-mir-130a-3p, hsa-mir-29c-3p, hsa-mir-301a-3p, hsa-mir-130b-3p, hsa-mir-30e-5p, hsa-mir-339-5p, hsa-mir-496, hsa-mir-520g-3p, hsa-mir-520h, hsa-mir-612, hsa-mir-661, hsa-mir-454-3p, hsa-mir-766-3p, hsa-mir-106a-3p, hsa-mir-196a-3p, hsa-mir-423-5p, hsa-mir-488-3p, hsa-mir-455-3p, hsa-mir-593-3p, hsa-mir-301b-3p, hsa-mir-1285-3p, hsa-mir-548l, hsa-mir-1266-5p, hsa-mir-1827, hsa-mir-3119, hsa-mir-3173-3p, hsa-mir-3065-5p, hsa-mir-4295, hsa-mir-4330, hsa-mir-3609, hsa-mir-3652, hsa-mir-3666, hsa-mir-3924, hsa-mir-4421, hsa-mir-4428, hsa-mir-4430, hsa-mir-548ah-5p, hsa-mir-3135b, hsa-mir-4518, hsa-mir-3187-5p, hsa-mir-3664-3p, hsa-mir-4680-5p, hsa-mir-203b-3p, hsa-mir-5189-5p, hsa-mir-5197-5p, hsa-mir-5699-3p, hsa-mir-1277-5p, hsa-mir-939-3p, hsa-mir-6504-3p, hsa-mir-6512-3p, hsa-mir-504-3p, hsa-mir-6720-5p, hsa-mir-6732-3p, hsa-mir-6736-3p, hsa-mir-6787-3p, hsa-mir-6800-3p, hsa-mir-6817-3p, hsa-mir-6818-3p, hsa-mir-6849-3p, hsa-mir-6860, hsa-mir-6873-3p, hsa-mir-6891-5p, hsa-mir-6895-3p, hsa-mir-7110-3p, hsa-mir-7158-3p, hsa-mir-1-3p, hsa-mir-147a, hsa-mir-603, hsa-mir-1913, hsa-mir-148b-5p, hsa-mir-4517, hsa-mir-571
RPL13A	hsa-mir-17-3p hsa-mir-26a-5p hsa-mir-31-5p hsa-mir-92a-3p hsa-mir-197-3p hsa-mir-10b-5p hsa-mir-181a-5p hsa-mir-181b-5p hsa-mir-181c-5p hsa-mir-23b-3p hsa-mir-124-3p hsa-mir-130a-3p hsa-mir-191-5p hsa-mir-149-5p hsa-mir-320a hsa-mir-34b-5p hsa-mir-301a-3p hsa-mir-130b-3p hsa-mir-331-3p hsa-mir-323b-5p hsa-mir-484 hsa-mir-181d-5p hsa-mir-519c-5p hsa-mir-518f-5p hsa-mir-526a hsa-mir-544a hsa-mir-552-3p hsa-mir-576-5p hsa-mir-769-3p hsa-mir-765 hsa-mir-196a-3p hsa-mir-214-5p hsa-mir-221-5p hsa-mir-138-2-3p hsa-mir-141-5p hsa-mir-125a-3p hsa-mir-423-5p hsa-mir-877-3p hsa-mir-301b-3p hsa-mir-523-5p hsa-mir-518e-5p hsa-mir-522-5p hsa-mir-519a-5p hsa-mir-519b-5p hsa-mir-520c-5p hsa-mir-518d-5p hsa-mir-103a-2-5p hsa-mir-3176 hsa-mir-3202 hsa-mir-4262 hsa-mir-4286 hsa-mir-3678-5p hsa-mir-3912-3p hsa-mir-3915 hsa-mir-3150b-3p hsa-mir-3928-3p hsa-mir-3934-5p hsa-mir-4476 hsa-mir-4649-5p hsa-mir-4685-5p hsa-mir-4689 hsa-mir-4710 hsa-mir-4742-5p hsa-mir-4777-5p hsa-mir-4436b-3p hsa-mir-4784 hsa-mir-4802-5p hsa-mir-766-5p hsa-mir-4632-5p hsa-mir-6071 hsa-mir-6508-5p hsa-mir-6514-3p hsa-mir-6717-5p hsa-mir-6721-5p hsa-mir-410-5p hsa-mir-494-5p hsa-mir-6729-5p hsa-mir-6735-5p hsa-mir-6738-3p hsa-mir-6811-3p hsa-mir-6828-3p hsa-mir-6837-5p hsa-mir-6858-5p hsa-mir-6876-5p hsa-mir-6879-5p hsa-mir-6894-5p hsa-mir-7113-5p hsa-mir-7154-3p hsa-mir-7843-5p hsa-mir-8067 hsa-mir-7977 hsa-mir-1-3p hsa-mir-134-5p hsa-mir-223-5p hsa-mir-376a-3p hsa-mir-376b-3p hsa-mir-595
RPL24	hsa-mir-22-3p hsa-mir-25-3p hsa-mir-32-5p hsa-mir-92a-3p hsa-mir-204-5p hsa-mir-150-5p hsa-mir-302a-5p hsa-mir-363-3p hsa-mir-367-3p hsa-mir-374a-5p hsa-mir-378a-5p hsa-mir-423-3p hsa-mir-433-3p hsa-mir-18a-3p hsa-mir-92b-3p hsa-mir-548c-3p hsa-mir-650 hsa-mir-186-3p hsa-mir-34b-3p hsa-mir-374a-3p hsa-mir-876-5p hsa-mir-374b-5p hsa-mir-940 hsa-mir-1224-3p hsa-mir-1234-3p hsa-mir-1976 hsa-mir-3122 hsa-mir-3178 hsa-mir-4325 hsa-mir-3612 hsa-mir-3614-5p hsa-mir-1273e hsa-mir-3913-5p hsa-mir-4454 hsa-mir-4468 hsa-mir-4668-5p hsa-mir-4695-5p hsa-mir-4727-5p hsa-mir-4743-5p hsa-mir-4746-3p hsa-mir-4756-3p hsa-mir-4768-3p hsa-mir-4769-3p hsa-mir-4792 hsa-mir-4798-3p hsa-mir-450a-

	1-3p hsa-mir-1277-5p hsa-mir-6499-5p hsa-mir-6500-3p hsa-mir-6508-5p hsa-mir-6511a-5p hsa-mir-6513-5p hsa-mir-383-3p hsa-mir-887-5p hsa-mir-1910-3p hsa-mir-6778-3p hsa-mir-6790-3p hsa-mir-6808-5p hsa-mir-6813-3p hsa-mir-6817-5p hsa-mir-6821-3p hsa-mir-6840-3p hsa-mir-6893-5p hsa-mir-7107-5p hsa-mir-7703 hsa-mir-8067 hsa-mir-7977 hsa-mir-3653-5p hsa-mir-145-5p hsa-mir-211-5p hsa-mir-603
RPS24	hsa-let-7a-5p hsa-let-7b-5p hsa-let-7c-5p hsa-let-7e-5p hsa-mir-16-5p hsa-mir-17-5p hsa-mir-26a-5p hsa-mir-27a-3p hsa-mir-92a-3p hsa-mir-96-5p hsa-mir-103a-3p hsa-mir-107 hsa-mir-129-5p hsa-mir-182-5p hsa-mir-210-3p hsa-mir-221-3p hsa-mir-23b-3p hsa-mir-27b-3p hsa-mir-128-3p hsa-mir-29c-3p hsa-mir-409-3p hsa-mir-483-3p hsa-mir-484 hsa-mir-511-5p hsa-mir-588 hsa-mir-609 hsa-mir-548c-3p hsa-mir-19b-1-5p hsa-mir-22-5p hsa-mir-33a-3p hsa-mir-10b-3p hsa-mir-140-3p hsa-mir-145-3p hsa-mir-877-5p hsa-mir-1301-3p hsa-mir-2116-5p hsa-mir-3125 hsa-mir-3152-3p hsa-mir-3065-3p hsa-mir-4317 hsa-mir-3616-3p hsa-mir-3681-3p hsa-mir-3916 hsa-mir-3927-3p hsa-mir-4451 hsa-mir-4477a hsa-mir-3677-5p hsa-mir-3973 hsa-mir-4668-3p hsa-mir-4677-5p hsa-mir-4694-5p hsa-mir-4701-5p hsa-mir-451b hsa-mir-4736 hsa-mir-4766-3p hsa-mir-4779 hsa-mir-4436b-3p hsa-mir-5047 hsa-mir-5693 hsa-mir-216a-3p hsa-mir-4632-5p hsa-mir-6721-5p hsa-mir-6735-5p hsa-mir-6804-5p hsa-mir-6828-5p hsa-mir-6831-5p hsa-mir-6859-5p hsa-mir-6879-5p hsa-mir-7156-3p hsa-mir-7843-5p hsa-mir-7974 hsa-mir-1-3p hsa-mir-9-5p hsa-mir-5009-5p hsa-mir-450a-5p hsa-mir-127-5p hsa-mir-200c-5p hsa-mir-628-3p hsa-mir-92a-1-5p
RPL37	hsa-mir-17-5p hsa-mir-26a-5p hsa-mir-26b-5p hsa-mir-32-5p hsa-mir-33a-5p hsa-mir-92a-3p hsa-mir-103a-3p hsa-mir-7-5p hsa-mir-34a-5p hsa-mir-181b-5p hsa-mir-23b-3p hsa-mir-122-5p hsa-mir-124-3p hsa-mir-137 hsa-mir-143-3p hsa-mir-191-5p hsa-mir-361-5p hsa-mir-373-5p hsa-mir-378a-5p hsa-mir-324-5p hsa-mir-338-3p hsa-mir-325 hsa-mir-433-3p hsa-mir-485-3p hsa-mir-494-3p hsa-mir-181d-5p hsa-mir-498 hsa-mir-455-5p hsa-mir-493-3p hsa-mir-92b-3p hsa-mir-562 hsa-mir-589-3p hsa-mir-616-5p hsa-mir-19a-5p hsa-mir-19b-1-5p hsa-mir-27a-5p hsa-mir-10b-3p hsa-mir-138-2-3p hsa-mir-140-3p hsa-mir-125a-3p hsa-mir-188-3p hsa-mir-106b-3p hsa-mir-130b-5p hsa-mir-371a-5p hsa-mir-338-5p hsa-mir-744-5p hsa-mir-935 hsa-mir-513c-5p hsa-mir-1283 hsa-mir-1297 hsa-mir-1976 hsa-mir-764 hsa-mir-2114-5p hsa-mir-3074-3p hsa-mir-3065-5p hsa-mir-514b-5p hsa-mir-4317 hsa-mir-4272 hsa-mir-4279 hsa-mir-3618 hsa-mir-3934-5p hsa-mir-4465 hsa-mir-4474-5p hsa-mir-4705 hsa-mir-4714-3p hsa-mir-4722-3p hsa-mir-371b-5p hsa-mir-4433a-5p hsa-mir-4482-3p hsa-mir-5010-3p hsa-mir-5011-5p hsa-mir-5571-5p hsa-mir-5588-3p hsa-mir-5697 hsa-mir-1304-3p hsa-mir-1277-5p hsa-mir-190a-3p hsa-mir-372-5p hsa-mir-1908-3p hsa-mir-6727-3p hsa-mir-6747-3p hsa-mir-6778-3p hsa-mir-6790-3p hsa-mir-6791-3p hsa-mir-6819-3p hsa-mir-6821-3p hsa-mir-6829-3p hsa-mir-6836-3p hsa-mir-6877-3p hsa-mir-7159-5p hsa-mir-7159-3p hsa-mir-3653-5p hsa-mir-126-3p hsa-mir-3929 hsa-mir-2467-5p hsa-mir-1307-5p hsa-mir-301a-5p hsa-mir-452-3p
RPS16	hsa-mir-17-3p hsa-mir-22-3p hsa-mir-24-3p hsa-mir-103a-3p hsa-mir-224-5p hsa-mir-149-5p hsa-mir-320a hsa-mir-373-5p hsa-mir-374a-5p hsa-mir-379-5p hsa-mir-381-3p hsa-mir-338-3p hsa-mir-424-5p hsa-mir-452-5p hsa-mir-497-5p hsa-mir-548a-3p hsa-mir-616-5p hsa-mir-627-5p hsa-mir-652-3p hsa-let-7a-3p hsa-let-7b-3p hsa-let-7f-1-3p hsa-let-7f-2-3p hsa-mir-19a-5p hsa-mir-19b-1-5p hsa-mir-27a-5p hsa-mir-145-3p hsa-mir-300 hsa-mir-876-5p hsa-mir-1296-5p hsa-mir-548e-3p hsa-mir-1303 hsa-mir-548f-3p hsa-mir-548g-3p hsa-mir-2052 hsa-mir-3159 hsa-mir-3167 hsa-mir-3174 hsa-mir-548x-3p hsa-mir-3613-3p hsa-mir-548ae-3p hsa-mir-548aj-3p hsa-mir-4490 hsa-mir-548am-3p hsa-mir-4666a-3p hsa-mir-4676-3p hsa-mir-371b-5p hsa-mir-4774-5p hsa-mir-4789-5p hsa-mir-4795-3p hsa-mir-548ah-3p hsa-mir-5010-3p hsa-mir-548aq-3p hsa-mir-548ar-3p hsa-mir-5702 hsa-mir-1185-2-3p hsa-mir-1185-1-3p hsa-mir-98-3p hsa-mir-937-5p hsa-mir-548az-3p hsa-mir-892c-3p hsa-mir-548j-3p hsa-mir-6882-5p hsa-mir-8055 hsa-mir-8081 hsa-mir-1-3p hsa-mir-216b-5p hsa-mir-1269a hsa-mir-150-3p hsa-mir-3661 hsa-mir-4511 hsa-mir-4804-5p hsa-mir-5009-5p hsa-mir-629-5p
RPL12	hsa-let-7b-5p hsa-let-7e-5p hsa-mir-16-5p hsa-mir-31-5p hsa-mir-100-5p hsa-mir-103a-3p hsa-mir-182-5p hsa-mir-218-5p hsa-mir-222-3p hsa-let-7g-5p hsa-mir-23b-3p hsa-mir-141-

	3p hsa-mir-191-5p hsa-mir-200a-3p hsa-mir-335-5p hsa-mir-494-3p hsa-mir-193b-3p hsa-mir-503-5p hsa-mir-539-5p hsa-mir-580-3p hsa-mir-612 hsa-mir-548c-3p hsa-mir-661 hsa-mir-769-5p hsa-mir-766-3p hsa-mir-33a-3p hsa-mir-488-3p hsa-mir-508-5p hsa-mir-1285-3p hsa-mir-4293 hsa-mir-3672 hsa-mir-3675-3p hsa-mir-4433a-3p hsa-mir-3187-5p hsa-mir-4648 hsa-mir-4654 hsa-mir-4684-5p hsa-mir-4747-5p hsa-mir-4769-5p hsa-mir-4786-5p hsa-mir-4793-3p hsa-mir-5189-5p hsa-mir-5196-5p hsa-mir-873-3p hsa-mir-1273g-3p hsa-mir-939-3p hsa-mir-1233-5p hsa-mir-6512-3p hsa-mir-134-3p hsa-mir-6720-5p hsa-mir-6771-3p hsa-mir-6778-5p hsa-mir-6849-3p hsa-mir-6860 hsa-mir-6864-3p hsa-mir-7703 hsa-mir-1-3p hsa-mir-190a-5p hsa-mir-181a-2-3p hsa-mir-214-3p
RPS27A	hsa-mir-17-5p hsa-mir-20a-5p hsa-mir-21-5p hsa-mir-26a-5p hsa-mir-29a-3p hsa-mir-93-5p hsa-mir-29b-3p hsa-mir-106a-5p hsa-mir-30c-5p hsa-mir-181a-5p hsa-mir-23b-3p hsa-mir-30b-5p hsa-mir-130a-3p hsa-mir-106b-5p hsa-mir-29c-3p hsa-mir-301a-3p hsa-mir-130b-3p hsa-mir-20b-5p hsa-mir-485-3p hsa-mir-519c-3p hsa-mir-526b-3p hsa-mir-519b-3p hsa-mir-519d-3p hsa-mir-520h hsa-mir-519a-3p hsa-mir-578 hsa-mir-643 hsa-mir-653-5p hsa-mir-454-3p hsa-mir-106a-3p hsa-mir-488-3p hsa-mir-665 hsa-mir-301b-3p hsa-mir-2681-5p hsa-mir-4295 hsa-mir-3666 hsa-mir-4649-3p hsa-mir-4722-5p hsa-mir-4756-3p hsa-mir-4768-3p hsa-mir-4797-5p hsa-mir-1273f hsa-mir-5011-5p hsa-mir-5681a hsa-mir-450a-1-3p hsa-mir-1277-5p hsa-mir-6716-5p hsa-mir-190a-3p hsa-mir-510-3p hsa-mir-651-3p hsa-mir-6730-5p hsa-mir-6885-3p hsa-mir-7160-5p hsa-mir-1-3p
RPS19	hsa-mir-16-5p hsa-mir-24-3p hsa-mir-10a-5p hsa-mir-10b-5p hsa-mir-142-3p hsa-mir-143-3p hsa-mir-191-5p hsa-mir-155-5p hsa-mir-34b-5p hsa-mir-501-5p hsa-mir-558 hsa-mir-566 hsa-mir-648 hsa-mir-652-3p hsa-mir-661 hsa-mir-766-3p hsa-mir-93-3p hsa-let-7g-3p hsa-mir-130b-5p hsa-mir-193b-5p hsa-mir-505-5p hsa-mir-508-5p hsa-mir-940 hsa-mir-941 hsa-mir-1285-3p hsa-mir-1827 hsa-mir-1976 hsa-mir-449c-5p hsa-mir-2682-5p hsa-mir-3160-3p hsa-mir-4252 hsa-mir-2355-5p hsa-mir-4477a hsa-mir-4486 hsa-mir-4487 hsa-mir-3677-5p hsa-mir-4649-5p hsa-mir-4708-5p hsa-mir-3591-5p hsa-mir-4770 hsa-mir-4793-3p hsa-mir-1273f hsa-mir-548at-5p hsa-mir-212-5p hsa-mir-1273g-3p hsa-mir-939-3p hsa-mir-6088 hsa-mir-6511a-5p hsa-mir-6512-3p hsa-mir-874-5p hsa-mir-1910-3p hsa-mir-6720-5p hsa-mir-6729-5p hsa-mir-6789-3p hsa-mir-6806-5p hsa-mir-6808-5p hsa-mir-6840-3p hsa-mir-6849-3p hsa-mir-6893-5p hsa-mir-7703 hsa-mir-1-3p hsa-mir-138-5p hsa-mir-4690-5p hsa-mir-1287-5p hsa-mir-1343-3p hsa-mir-185-3p hsa-mir-3158-3p hsa-mir-320e hsa-mir-660-5p
RPL27A	hsa-let-7b-5p hsa-let-7e-5p hsa-mir-15a-5p hsa-mir-16-5p hsa-mir-17-3p hsa-mir-19a-3p hsa-mir-19b-3p hsa-mir-26a-5p hsa-mir-30a-3p hsa-mir-31-5p hsa-mir-92a-3p hsa-mir-93-5p hsa-mir-196a-5p hsa-mir-30c-5p hsa-mir-139-5p hsa-mir-34a-5p hsa-mir-15b-5p hsa-mir-23b-3p hsa-mir-124-3p hsa-mir-191-5p hsa-mir-195-5p hsa-mir-155-5p hsa-mir-99b-5p hsa-mir-378a-5p hsa-mir-342-3p hsa-mir-484 hsa-mir-485-5p hsa-mir-490-3p hsa-mir-494-3p hsa-mir-193b-3p hsa-mir-498 hsa-mir-501-5p hsa-mir-503-5p hsa-mir-556-5p hsa-mir-658 hsa-mir-766-3p hsa-mir-93-3p hsa-mir-29b-2-5p hsa-mir-221-5p hsa-mir-186-3p hsa-mir-99b-3p hsa-mir-296-3p hsa-mir-130b-5p hsa-mir-361-3p hsa-mir-500a-5p hsa-mir-508-5p hsa-mir-1227-3p hsa-mir-513c-5p hsa-mir-1180-3p hsa-mir-548l hsa-mir-1468-5p hsa-mir-2114-3p hsa-mir-3128 hsa-mir-514b-5p hsa-mir-4252 hsa-mir-4459 hsa-mir-4490 hsa-mir-4537 hsa-mir-4691-3p hsa-mir-4726-3p hsa-mir-4774-5p hsa-mir-4793-3p hsa-mir-873-3p hsa-mir-1273g-3p hsa-mir-6499-3p hsa-mir-6512-3p hsa-mir-6720-5p hsa-mir-6809-5p hsa-mir-6823-3p hsa-mir-6849-3p hsa-mir-6884-5p hsa-mir-7703 hsa-mir-8073 hsa-mir-28-5p hsa-mir-1-3p hsa-mir-4685-3p hsa-mir-126-3p hsa-mir-326 hsa-mir-624-3p hsa-mir-15a-3p hsa-mir-362-5p hsa-mir-374b-3p hsa-mir-196b-5p hsa-mir-146a-5p hsa-mir-3929 hsa-mir-214-3p hsa-mir-151a-5p hsa-mir-1179 hsa-mir-126-5p hsa-mir-202-5p hsa-mir-299-3p hsa-mir-345-5p hsa-mir-4738-3p hsa-mir-500b-5p hsa-mir-548a-5p hsa-mir-548am-5p hsa-mir-548b-5p hsa-mir-548c-5p hsa-mir-548d-5p hsa-mir-548h-5p hsa-mir-548i hsa-mir-548j-5p hsa-mir-548k hsa-mir-548n hsa-mir-548o-5p hsa-mir-548w hsa-mir-548y hsa-mir-647 hsa-mir-203a-3p
RPL23A	hsa-mir-23a-3p hsa-mir-27a-3p hsa-mir-33a-5p hsa-mir-148a-3p hsa-mir-23b-3p hsa-mir-27b-3p hsa-mir-124-3p hsa-mir-191-5p hsa-mir-484 hsa-mir-485-3p hsa-mir-487a-3p hsa-

	mir-193b-3p hsa-mir-526b-5p hsa-mir-503-5p hsa-mir-545-3p hsa-mir-609 hsa-mir-641 hsa-mir-411-5p hsa-mir-671-5p hsa-mir-769-3p hsa-mir-33a-3p hsa-mir-214-5p hsa-mir-125b-2-3p hsa-mir-423-5p hsa-mir-877-5p hsa-mir-1224-5p hsa-mir-3126-5p hsa-mir-3127-5p hsa-mir-3166 hsa-mir-4316 hsa-mir-3650 hsa-mir-3936 hsa-mir-4419a hsa-mir-4455 hsa-mir-4510 hsa-mir-4520-3p hsa-mir-4698 hsa-mir-4751 hsa-mir-4520-2-3p hsa-mir-5004-5p hsa-mir-5011-5p hsa-mir-506-5p hsa-mir-1277-5p hsa-mir-6074 hsa-mir-6127 hsa-mir-6129 hsa-mir-6130 hsa-mir-6133 hsa-mir-892c-5p hsa-mir-190a-3p hsa-mir-6772-5p hsa-mir-6782-5p hsa-mir-6809-5p hsa-mir-6818-5p hsa-mir-6828-5p hsa-mir-6834-5p hsa-mir-6875-5p hsa-mir-8076 hsa-mir-9500 hsa-mir-129-2-3p hsa-mir-1-3p hsa-mir-34a-3p hsa-mir-9-5p hsa-mir-140-5p hsa-mir-203a-3p hsa-mir-138-1-3p hsa-mir-148b-3p hsa-mir-33b-5p hsa-mir-4326 hsa-mir-499a-3p hsa-mir-5094 hsa-mir-561-5p hsa-mir-574-5p
RPLP0	hsa-mir-16-5p hsa-mir-17-3p hsa-mir-22-3p hsa-mir-29a-3p hsa-mir-29b-3p hsa-mir-103a-3p hsa-mir-30d-5p hsa-mir-7-5p hsa-mir-181a-5p hsa-mir-181b-5p hsa-mir-181c-5p hsa-mir-183-5p hsa-mir-221-3p hsa-mir-15b-5p hsa-mir-125b-5p hsa-mir-149-5p hsa-mir-186-5p hsa-mir-155-5p hsa-mir-29c-3p hsa-mir-30e-5p hsa-mir-382-5p hsa-mir-340-3p hsa-mir-330-3p hsa-mir-331-3p hsa-mir-324-3p hsa-mir-484 hsa-mir-494-3p hsa-mir-193b-3p hsa-mir-497-5p hsa-mir-181d-5p hsa-mir-527 hsa-mir-502-5p hsa-mir-503-5p hsa-mir-505-3p hsa-mir-507 hsa-mir-92b-3p hsa-mir-557 hsa-mir-548a-3p hsa-mir-671-5p hsa-mir-19a-5p hsa-mir-19b-1-5p hsa-mir-19b-2-5p hsa-mir-20a-3p hsa-mir-140-3p hsa-mir-450b-5p hsa-mir-892b hsa-mir-744-3p hsa-mir-522-5p hsa-mir-518a-5p hsa-mir-548e-3p hsa-mir-548f-3p hsa-mir-664a-5p hsa-mir-2052 hsa-mir-3680-3p hsa-mir-4794 hsa-mir-548ar-3p hsa-mir-5582-3p hsa-mir-548aw hsa-mir-495-5p hsa-mir-548az-3p hsa-mir-129-2-3p hsa-mir-628-5p hsa-mir-362-3p hsa-mir-3685 hsa-mir-3689a-5p hsa-mir-3689b-5p hsa-mir-3689e hsa-mir-3689f hsa-mir-432-5p hsa-mir-5581-5p hsa-mir-887-3p
